# Dichlorvos-induced formation of isopeptide crosslinks between proteins in SH-SY5Y cells

**DOI:** 10.1016/j.ab.2022.114844

**Published:** 2022-08-09

**Authors:** Lawrence M. Schopfer, Oksana Lockridge

**Affiliations:** Eppley Institute, University of Nebraska Medical Center, Omaha, NE, 68198, USA

**Keywords:** Isopeptide, Crosslinks, SH-SY5Y human Neuroblastoma cells, Dichlorvos, Protein Prospector, Mass spectrometry, Crosslink, Manual evaluation

## Abstract

Chlorpyrifos oxon catalyzes the crosslinking of proteins via an isopeptide bond between lysine and glutamic acid or aspartic acid in studies with purified proteins. Our goal was to determine the crosslinking activity of the organophosphorus pesticide, dichlorvos. We developed a protocol for examining crosslinks in a complex protein mixture consisting of human SH-SY5Y cells exposed to 10 μM dichlorvos. The steps in our protocol included immunopurification of crosslinked peptides by binding to *anti*-isopeptide antibody 81D1C2, stringent washing of the immobilized complex, release of bound peptides from Protein G agarose with 50% acetonitrile 1% formic acid, liquid chromatography tandem mass spectrometry on an Orbitrap Fusion Lumos mass spectrometer, Protein Prospector searches of mass spectrometry data, and manual evaluation of candidate crosslinked dipeptides. We report a low quantity of dichlorvos-induced KD and KE crosslinked proteins in human SH-SY5Y cells exposed to dichlorvos. Cells not treated with dichlorvos had no detectable KD and KE crosslinked proteins. Proteins in the crosslink were low abundance proteins. In conclusion, we provide a protocol for testing complex protein mixtures for the presence of crosslinked proteins. Our protocol could be useful for testing the association between neurodegenerative disease and exposure to organophosphorus pesticides.

## 1. Introduction

Formation of γ-glutamyl-ε-lysine isopeptide bonds between the ε-amine of lysine and γ-glutamyl of glutamine is mediated by the transamidase activity of transglutaminase, a family of enzymes that includes fibrin stabilizing factor XIII [1].

The earliest method for detecting isopeptide crosslinks used extensive proteolysis followed by amino acid composition analysis [1,2] or high-pressure liquid chromatography [3] to identify the γ-glutamyl-ε-lysine product. More recently, isopeptide crosslinks were detected by *anti*-isopeptide antibodies in immunohistochemically stained brain sections and in Western blots. None of these methods was capable of identifying the peptides that gave rise to the reactive lysine and glutamine or the specific residues that were crosslinked. Using amino acid sequencing after reaction with a radiolabeled glutamine substrate and limited proteolysis provided a means for identifying the labeled protein/peptide. However, a variety of artifacts limits this technique to proteins that have already been shown to be substrates for transglutaminase [3]. Mass spectrometry offers a more robust means for studying isopeptide crosslinked peptides because it can identify the specific amino acids, peptides, and proteins involved. Nemes and coworkers demonstrated the power of mass spectrometry by identifying 2 proteins from brain cortex that were crosslinked to ubiquitin via lysine-glutamine isopeptide bonds and an intramolecular crosslink between Gln99 and Lys58 of α-synuclein [4]. The amount of isopeptide crosslinked peptides was so low that to detect them the preparations had to be highly enriched, by immunopurification with *anti*-isopeptide antibodies [4].

**Fig. 1.**
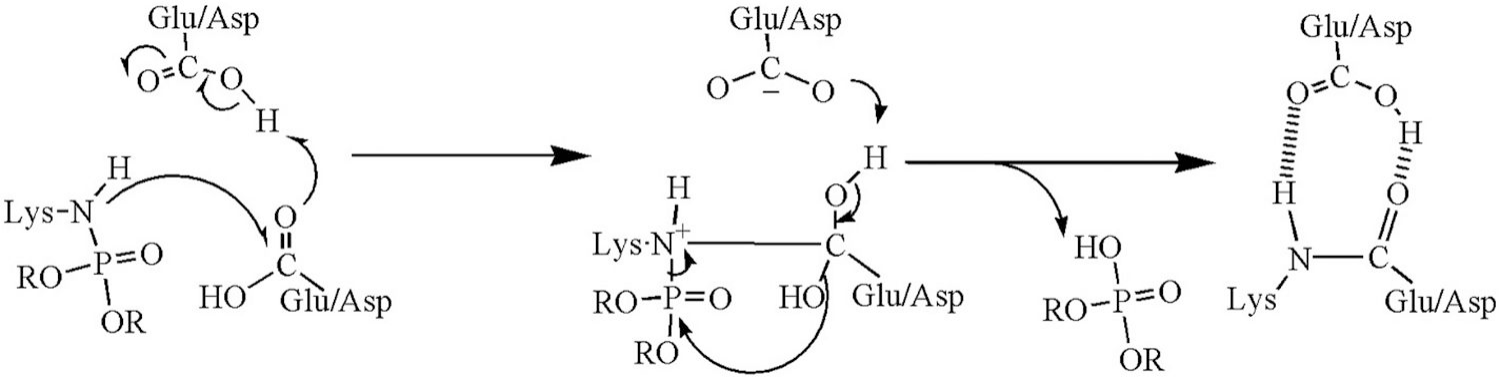
Mechanism for organophosphate-induced isopeptide bond formation. The structure on the left shows organophosphate covalently attached to the epsilon amino group of lysine. Attack of the lysine amine on the Glu/Asp carbonyl carbon is catalyzed by a vicinal acidic group. The middle structure shows an intermediate between the organophosphate-modified lysine and the side chain of glutamic or aspartic acid. In the last step, the organophosphate is released and a covalent isopeptide bond forms between lysine and glutamic or aspartic acid. A nearby acidic residue stabilizes the crosslink. This mechanism is analogous to that for spontaneous formation of isopeptide bonds proposed by Kang et al. [8]. Mass spectrometry distinguishes chemically induced crosslinks from transglutaminase catalyzed crosslinks by identifying the crosslinked residues and the peptides in the crosslink. Reproduced by permission [9].

Mass spectrometry has been used to explore lysine-glutamate and lysine-aspartate isopeptide crosslinks induced by the organophosphorus pesticide chlorpyrifos oxon. Crosslinked peptides were found for tubulin, and butyrylcholinesterase [5,6]. The organophosphorus nerve agent VX induced isopeptide crosslinks on ubiquitin [7]. A proposed mechanism for isopeptide bond formation catalyzed by organophosphate chemicals is shown in Fig. 1.

Mass spectrometry can distinguish chemically-induced cross-links from transglutaminase-mediated cross-links because the identity of the cross-linked residues can be determined. The sequence of the peptides is part of the result. Thus the residues involved in the crosslink can be identified, making it possible to distinguish a Gln-Lys crosslink mediated by transglutaminase from a Glu-Lys crosslink induced by organophosphate. In addition, the Protein Prospector/Batch Tag database search parameter “link aa” stipulates that the allowed amino acids in the cross-link are “E, D, protein C-term → K, protein N-term” thereby excluding Gln, and the search parameter “bridge comp” stipulates that the allowed delta mass for the crosslink is H_2_O (−18 Da), where transglutaminase-mediated crosslinks would require “link aa” to be Q → K with a “bridge comp” of NH_3_ (−17 Da).

Previous studies were performed on pure proteins. A study of organophosphorus-induced isopeptide crosslinking in microtubule-associated protein complex demonstrated that complex protein preparations are amenable to analysis [10].

In the present report, we have expanded our use of mass spectrometry to identify isopeptide crosslinked peptides from a more complex source. Human SH-SY5Y neuroblastoma cells were treated with the organophosphorus pesticide dichlorvos. Cells were lysed and digested with trypsin. Isopeptide crosslinked peptides were enriched with the *anti*-isopeptide antibody 81D1C2 and subjected to liquid chromatography-tandem mass spectrometry. Mass spectral data were analyzed with Protein Prospector/Batch Tag Web and Xcalibur/Qual Browser. In the course of analyzing these data, we have developed a generalized protocol.

## 2. Experimental

### 2.1. Chemicals

Human neuroblastoma SH-SY5Y cells (American Type Culture Collection #CRL-2266).

Trypsin (Promega, Sequencing grade, #V5113) stored at −80 °C.

Dichlorvos CAS 62-73-7 (Chem Service Inc. #N11675) 0.01 M stock solution in acetonitrile stored at −80 °C.

DMEM/F12 GlutaMAX (Gibco #10565–108).

Fetal bovine serum (Life Tech #16000044).

Penicillin & streptomycin (Gibco #15140–122).

Trans retinoic acid (Sigma-Aldrich #554720).

Pierce IP lysis buffer (25 mM TrisCl pH 7.4, 150 mM NaCl, 1% NP-40, 1 mM EDTA, 5% glycerol) (Thermo Scientific #87787).

Halt protease inhibitor cocktail (Thermo Fisher Scientific #78430); containing AEBSF, aprotinin, bestatin, E–64, leupeptin and pepstatin A.

RIPA buffer (25 mM Tris-HCl pH 7.6, 1% NP-40, 1% sodium deoxycholate, 0.1% SDS, 140 mM NaCl) (Thermo Fisher Scientific #89900).

Mouse *anti*-isopeptide monoclonal 81D1C2 (LS Bio #LS-C153331); reconstituted with water to 1 mg/mL.

Protein G agarose (Protein Mods LLC #PGGH); binds 20 mg antibody per ml beads.

Bicinchoninic acid protein assay kit (Thermo Scientific #23228).

### 2.2. Cell culture

SH-SY5Y cells (ATCC CRL #2266) in T75 flasks were grown in DMEM/F12 GlutaMAX supplemented with 15% fetal bovine serum, penicillin, and streptomycin, in a humidified atmosphere of 5% carbon dioxide, at 37 °C. After 5 days, when cells were 70–80% confluent, cells were washed with phosphate buffered saline (PBS). Cells received 15 mL per flask of DMEM/F12 GlutaMAX (no serum) supplemented with 10 μM trans retinoic acid plus-or-minus 10 μM dichlorvos. After 2 days at 37 °C, cells were harvested.

### 2.3. Cell lysis and protein concentration

Cells were harvested, washed with PBS, and lysed with 100 μL IP lysis buffer (25 mM TrisCl pH 7.4, 150 mM NaCl, 1% NP-40, 1 mM EDTA, 5% glycerol) containing Halt protease inhibitor cocktail. Halt protease inhibitor cocktail contains AEBSF (4-(2-aminoethyl)-benzene sulfonyl fluoride), aprotinin, bestatin, E–64 (epoxysuccinyl-l-leucylamido (4-guandidino) butane), leupeptin and pepstatin A, that inhibit serine-proteases, cysteine proteases, aspartic acid proteases, and aminopeptidases. Cell debris was removed by centrifugation at 14,000×*g* for 20 min at 4 °C. The protein concentration in 130 μL of supernatant was 13.2 mg/mL as determined by the bicinchoninic acid protein assay (Thermo Scientific #23228).

Three cell lysates were prepared for the plus dichlorvos cells and two for the minus dichlorvos cells. Each lysate was digested with trypsin, immunopurified with *anti*-isopeptide antibody 81D1C2, and subjected to liquid chromatography tandem mass spectrometry.

### 2.4. Trypsin digestion

Cell lysate supernatant containing 200 μg protein (15 μL) was diluted with 185 μL of 20 mM ammonium bicarbonate pH 8. Proteins were denatured in a boiling water bath for 3 min. The denatured proteins were digested with 4 μg trypsin (8 μL) at 37 °C for 16 h without reduction and alkylation. Trypsin was inactivated by heating the digest in a boiling water bath for 3 min.

### 2.5. Immunopurification of tryptic peptides linked through an isopeptide bond

The heat-treated digest was incubated with 8 μg (8 μL) of *anti*-isopeptide monoclonal 81D1C2 at room temperature for 8 h to capture isopeptide crosslinked peptides. Antibody-peptide complexes were immobilized by adding 0.1 mL of a 1:1 suspension of Protein G agarose beads, in PBS. The sample was rotated overnight at room temperature.

The beads and liquid were transferred to a 0.45 μm Durapore spin filter (Millipore UFC30HV00). Use of the spin filter maximized recovery because beads were not lost in the wash steps. Beads were washed with 0.4 mL of RIPA buffer (25 mM Tris-HCl pH 7.6, 1% NP-40, 1% sodium deoxycholate, 0.1% SDS, 140 mM NaCl) 5 times followed by 5 washes with water. Salts and detergents were washed off with water. The flow through in each wash step was discarded.

The basket of washed beads was transferred to a new microfuge tube. Bound peptides were released from the washed beads by incubating the basket of beads with 0.1 mL of 50% acetonitrile, 1% formic acid for 0.5–1 h at room temperature. The released peptides were collected in the flow through by brief centrifugation. The extraction step was repeated twice. The combined flow through was dried by vacuum centrifugation.

### 2.6. Sample preparation for mass spectrometry

The dry sample was dissolved in 20 μL water. The sample was centrifuged for 30 min at 14,000×*g* and 4 °C. The top 10 μL were transferred to an autosampler vial.

### 2.7. Mass spectral data acquisition

Peptide separation was performed with a Thermo RSLC Ultimate 3000 ultra-high pressure liquid chromatography system (Thermo Scientific) at 36 °C. Solvent A was 0.1% formic acid in water, and solvent B was 0.1% formic acid in 80% acetonitrile. Peptides were loaded onto an Acclaim PepMap 100C18 trap column (75 μm × 2 cm; Thermo Scientific cat# 165535) at a flow rate of 4 μL/min and washed with 98% solvent A/2% solvent B for 10 min. Then, they were transferred to a Thermo Easy-Spray PepMap RSLC C18 column (75 μm × 50 cm with 2 μm particles, Thermo Scientific cat# ES803) and separated at a flow rate of 300 nL/min using a gradient of 9–50% solvent B in 30 min, 50–99% solvent B in 40 min, hold at 99% solvent B for 10 min, 99 to 9% solvent B in 4 min, hold at 9% solvent B for 16 min.

Eluted peptides were sprayed directly into a Thermo Orbitrap Fusion Lumos Tribrid mass spectrometer (Thermo Scientific). Data were collected using data dependent acquisition. A survey full scan MS (from *m/z* 350–1800) was acquired in the Orbitrap with a resolution of 120,000. The AGC target (Automatic Gain Control for setting the ion population in the Orbitrap before collecting the MS) was set at 4 × 10^5^ and the ion filling time was set at 50 msec. The 25 most intense ions with charge state of 2–6 were isolated in a 3 s cycle and fragmented using high-energy collision induced dissociation with 35% normalized collision energy. Fragment ions were detected in the Orbitrap with a mass resolution of 30,000 at m/z 200. The AGC target for MS/MS was set at 5 × 10^4^, and dynamic exclusion was set at 30 s with a 10 ppm mass window. Data were reported in *.raw format.

The *.raw data files were converted to *.mgf files using MSConvert (ProteoWizard Tools from SourceForge).

### 2.8. Batch Tag Web search for crosslinked peptide candidates

The *.mgf files were subjected to a database search using the Batch Tag Web algorithm in Protein Prospector version 6.2.1. Searches were performed on the Protein Prospector website https://prospector.ucsf.edu [prospector.ucsf.edu] (14May2022). Database search parameters included: database—SwissProt 2021.0618; Species—Homo sapiens; enzyme—trypsin, missed cleavages—3; expect calc method—none; protein N-term—unchecked; protein C-term—unchecked; uncleaved—checked; parent mass tolerance—20 ppm; fragment mass tolerance—30 ppm; precursor charge state—2, 3, 4, 5; parent ion conversion—monoisotopic; modification defect—0.0048 Da; instrument—ESI Q high res; link search type—user defined link; link aa—E, D, protein C-term→ K, protein N-term; mod comp ion—K, D, and E; mod range– −18 to 3883 Da; bridge comp—H-2O-1; mod uncleaved—checked; msms mass peaks—80; msms max modifications—2; variable modification—oxidation methionine; fixed modification—none. This database search created a list of peptides that Protein Prospector considered to be crosslinked. The list of potentially crosslinked peptides, along with parameters indicating the level of confidence in the assignment, were displayed in Protein Prospector/Search Compare.

Note that using smaller mass tolerances for parent mass and fragment mass did not improve the detection of isopeptide crosslinked peptides.

### 2.9. Search Compare screening of crosslinked peptide candidates

To reduce the number of crosslink peptide candidates and aid in the identification of crosslinked peptides, the Search Compare list was screened using the Protein Prospector output parameters. Parameters indicating a crosslinked peptide were taken to be: charge state 3, 4, 5; Score >25; score difference >1; % matched intensity >45%; and at least 5 amino acids in each peptide. Choice of these parameters is empirical and was based on experience.

### 2.10. Manual evaluation of crosslinked peptide candidates

Ultimately, crosslinked peptides were confirmed by manual evaluation. Discussion of the manual evaluation that we employed requires an understanding of the following terms:

1. A crosslink candidate is a pair of crosslinked peptides selected by Protein Prospector from a database search.
2. A crosslink specific mass is a mass in the MS/MS spectrum from a crosslink candidate that includes residues from both peptides.
3. A crosslink specific amino acid is an interval in the MS/MS spectrum from a crosslink candidate that is defined by two crosslink specific masses and corresponds to an amino acid that is part of the crosslink candidate sequence.
4. A ladder sequence is a term used by Protein Prospector to describe neutral loss of amino acids from the N-terminus of the parent ion. Ladder sequencing losses can occur from both peptides in the same MS/MS spectrum.
5. A peeling sequence is a term used by Protein Prospector to describe the neutral loss of an amino acid from the C-terminus of the parent ion. This is otherwise referred to as a [b_n-1_ + 18] fragment [11]. Any C-terminal residue can be lost, provided that a basic residue such as arginine, lysine or histidine is present in the sequence [12].
6. Mixed fragmentation refers to a series of crosslink specific masses that correspond to sequential removal of amino acids from both peptides in the crosslink candidate.
7. Peptide rearrangement consists of the transfer of amino acid(s) from one terminus of a peptide to the other. Rearrangement sometimes was helpful in mixed fragmentation scenarios. Two mechanisms have been described to explain peptide rearrangements. One is protease induced cyclization and ring re-opening during proteolysis. The rearrangement is believed to require a missed endo-proteinase cleavage site (i.e., an arginine or lysine for trypsinolysis) within 2-residues of either terminus [13]. The other mechanism for peptide rearrangement occurs in the mass spectrometer. Rearrangement occurs from linear b-ions by cyclization and subsequent ring opening. The cyclic form can open at various amide bonds effectively shifting residues from one terminus to the other [14].

### 2.11. Manual evaluation criteria that support the presence of crosslinked peptides

For a crosslink candidate to be accepted as a crosslinked peptide there must be amino acid sequence support for both peptides and there must be at least one crosslink specific amino acid, defined by two crosslink specific ions. Sequence support consists of the following features.

**Fig. 2.**
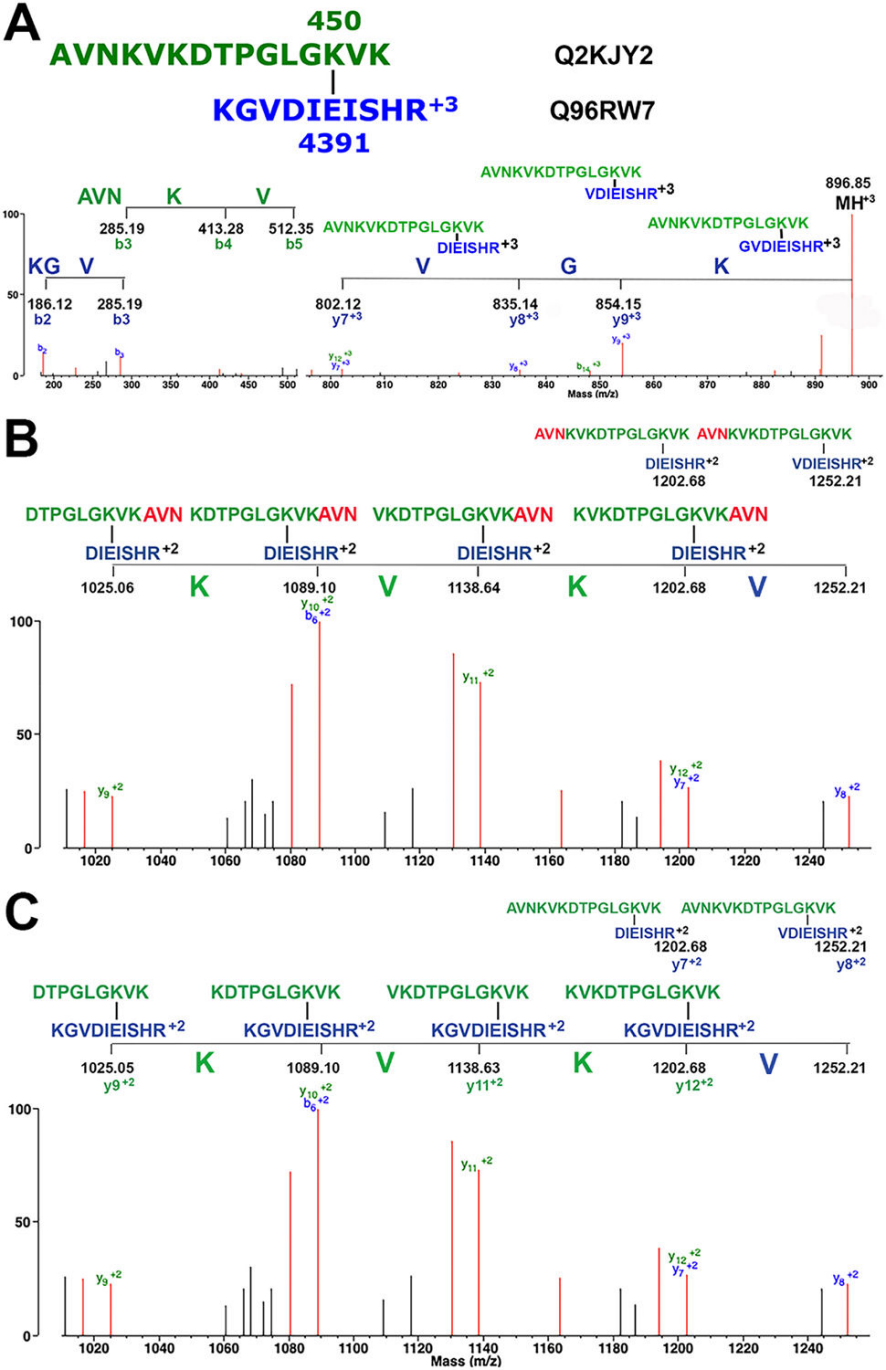
The MS/MS spectrum for the isopeptide crosslinked peptide AVNKVKDTPGLGK_450_VK/KGVDIE_4391_ISHR, where the subscripted residues indicate the site of the isopeptide crosslink. The parent ion is at *m/z* 896.85, +3 charged. Panel A shows the mass range from *m/z* 180 to 900. This includes a 5-amino acid, +1, b-ion sequence (AVNKV) from the N-terminal of the green peptide, a 3-amino acid, +1, b-ion sequence (KGV) from the N-terminal of the blue peptide, and a 3-amino acid, +3, ladder sequence (KGV) from the parent ion. Panel B shows the mass range *m/z* 1010 to 1260. This illustrates the first scenario for the mixed fragmentation. Structures of the proposed fragments are shown. Panel C also shows the mass range *m/z* 1010 to 1260. This illustrates the second scenario for the mixed fragmentation. Structures of the proposed fragments are shown. Unlabeled, red masses mostly represent loss of water, amine, or CO.

**Fig. 3.**
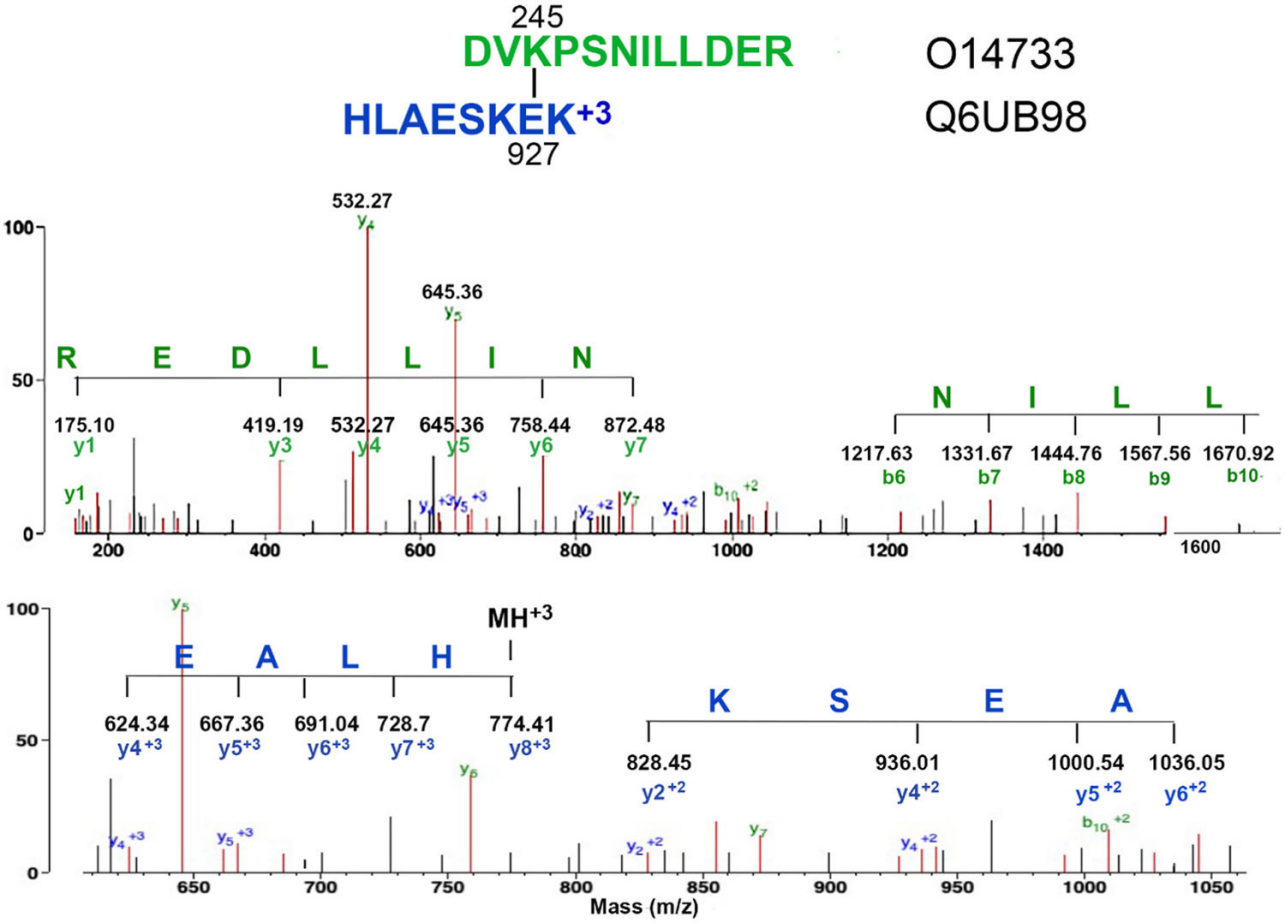
The MS/MS spectrum for the isopeptide crosslinked peptide DVK_245_PSNILLDER/HLAESKE_927_K, where the subscripted residues indicate the site of the isopeptide crosslink. The parent ion is at *m/z* 774.41, +3 charge. The upper panel shows the complete mass range *m/z* 220 to 1300. This shows a 7-amino acid, +1, y-ion sequence (NILLDER) from the green peptide and a 4-amino acid, +1, b-ion crosslink specific sequence (NILL) from the green peptide. The lower panel shows an expanded mass range from *m/z* 610 to 1060. This emphasizes a 4-amino acid, +3, ladder sequence (HLAE) from the blue peptide, and a 4-amino acid, +2 y-ion crosslink specific sequence (AESK) from the blue peptide. Unlabeled, red masses mostly represent loss of water, amine, or CO.

1. A series of non-crosslink specific masses in the MS/MS spectrum must correspond to an amino acid sequence from one or the other peptide in a crosslink candidate. Suitable sequences include an N-terminal sequence, a C-terminal sequence, or an internal fragment. Sequences must be at least 3 amino acids long (for example green AVNKV and blue KGV in Fig. 2 panel A and green REDLLIN in Fig. 3 upper panel).
2. At least one crosslink specific amino acid is essential. A series of crosslink specific amino acids is frequently encountered.
  2a) Sometimes the entire series consists of crosslink specific amino acids from one peptide. This is taken as strong evidence for a crosslinked peptide (for example blue KSEA in Fig. 3 lower panel).
  2b) Sometimes a series of crosslink specific amino acids will be appended by a single amino acid that is not part of the crosslink candidate. Such a sequence is still accepted as support for a crosslinked peptide (for example an unassigned V residue is appended to blue KV in Fig. 7 upper panel).
  2c) Sometimes a series of crosslink specific amino acids will be appended by more than one amino acid that is not part of the crosslink candidate. Such a sequence is not accepted (for example unassigned KV is appended to blue V in Fig. 6 upper panel).
  2d) Sometimes the entire series consists of a mixture of crosslink specific amino acids from both peptides, or of a mixture of amino acids from both the N- and C-terminals of one peptide. We refer to this as mixed fragmentation. This is taken as support for a crosslinked peptide (see Fig. 2, panels B and C, Fig. 6 lower panel, Fig. 7 lower panel for examples). Occasionally, mixed fragmentation can require rearrangement of a peptide sequence by shifting residues from the N-terminus to the C-terminus, or vice-versa (see Fig. 2, panel B for an example).
  2e) Sometimes a sequence of amino acids can be identified that is unrelated to the crosslinked candidate even though it may contain a few crosslinked specific amino acids. Such a sequence is rejected as support for the crosslink (for example QFLELY in Fig. 5 lower panel).
3. Neutral loss of amino acids from the parent ion. Neutral losses can be N-terminal amino acids (ladder sequence) from one peptide (for example blue VGK in Fig. 2 panel A, blue EALH in Fig. 3 lower panel, green VGK in Fig. 7 upper panel), C-terminal amino acids (peeling sequence) from one peptide, a combination of N-terminal and C-terminal amino acids from one peptide, or a mixture of N-terminal and C-terminal amino acids from both peptides. By definition, the amino acids that are neutral losses from the parent ion contain residues from both peptides and are therefore crosslink specific amino acids.

## 3. Results

### 3.1. Isopeptide crosslinked peptides

Three separate preparations of dichlorvos-treated SH-SY5Y cells were analyzed for isopeptide crosslinks between lysine and glutamate or lysine and aspartate. Forty isopeptide crosslinks were confirmed by manual evaluation. The results are given in Table 1. Two separate preparations of SH-SY5Y cells were made without exposure to dichlorvos. No isopeptide crosslinks between lysine and glutamate or lysine and aspartate were detected in cells not treated with dichlorvos.

All data were obtained from the soluble fraction of the cell lysate, therefore proteins in the aggregated pellet were not evaluated. Though the pellet could contain aggregated proteins that might be of interest, the complexity of the pellet made working with it unappealing. In our experience, aggregated proteins are soluble and remain in the cell lysate supernatant.

### 3.2. Peptide abundance

Only two crosslinked peptides appeared more than once. The crosslinked peptide AVNKVKDTPGLGK_450_VK/KGVDIE_4391_ISHR from protein kinesin-like protein KIF26B and hemicentin-1 appeared 5-times: twice in the first repetition (charge states 3 and 4), once in the second repetition, and twice in the third repetition (charge states 4 and 5). See Table 1.

The crosslinked peptide VTKKE_97_TLKAQK/TVGAAQLK_2180_PTLNQ LKQTQK from oxysterol-binding protein-related protein 5 and serine/threonine-protein kinase WNK2 appeared twice, once in the first repetition and once in the second repetition.

We attribute this low degree of reproducibility to crosslinking between low abundance proteins. To test this assumption, we compared the crosslinked proteins to two databases where the cellular concentration of the proteins was determined, see Supplementary Material Table S2.

Beck and coworkers determined the protein abundance for 7311 proteins from the human osteosarcoma cell line U20S [15]. Protein abundance ranged from <5 × 10^2^ to 6.53 × 10^6^ copies per cell. Abundance determination was based on 144 heavy isotope labeled reference peptides. The abundance of individual proteins in our crosslinked pairs was similar, ranging from <5 × 10^2^ to 4.42 × 10^6^ (see Supplementary Material Table S2). However, one member of each crosslinked pair was always from a low abundance protein (abundance less than 8 × 10^3^ or 0.1% of the maximum).

Geiger and coworkers determined the protein abundance for eleven cell lines (A549, GAMG, HEK293, HeLa, HepG2, K562, Jurkat, LnCap, MCF7, RKO, and U20S) [16]. A total of 11,732 proteins were detected. Proteins were reported as iBAQ values (intensity based absolute quantitation). iBAQ values are obtained by summing the peak intensities for all peptides matching to a specific protein and dividing by the number of theoretically observable peptides in the sample. iBAQ values are reported as log10 [17]. Protein abundance in the database ranged from 2.41 to 9.13. The abundance of individual proteins for our crosslinked pairs spanned a similar range, 2.42 to 8.78 (see Supplementary Material Table S2). However, one member of each crosslinked pair was always from a low abundance protein (abundance of less than 5 or 0.01% of the maximum).

The fact that most of the crosslinked peptides are from low abundance proteins illustrates one difficulty in obtaining isopeptide crosslinking data, low abundance proteins usually give low signals in the mass spectrometer. This also makes the frequency at which the crosslink occurs difficult to determine. Normally, we would use the number of times a given spectrum appears in the data (spectral count) as a measure of frequency. Most of the crosslinks in this data appear only once.

### 3.3. Examples of MS/MS spectra for isopeptide crosslinked peptides

**Fig. 4.**
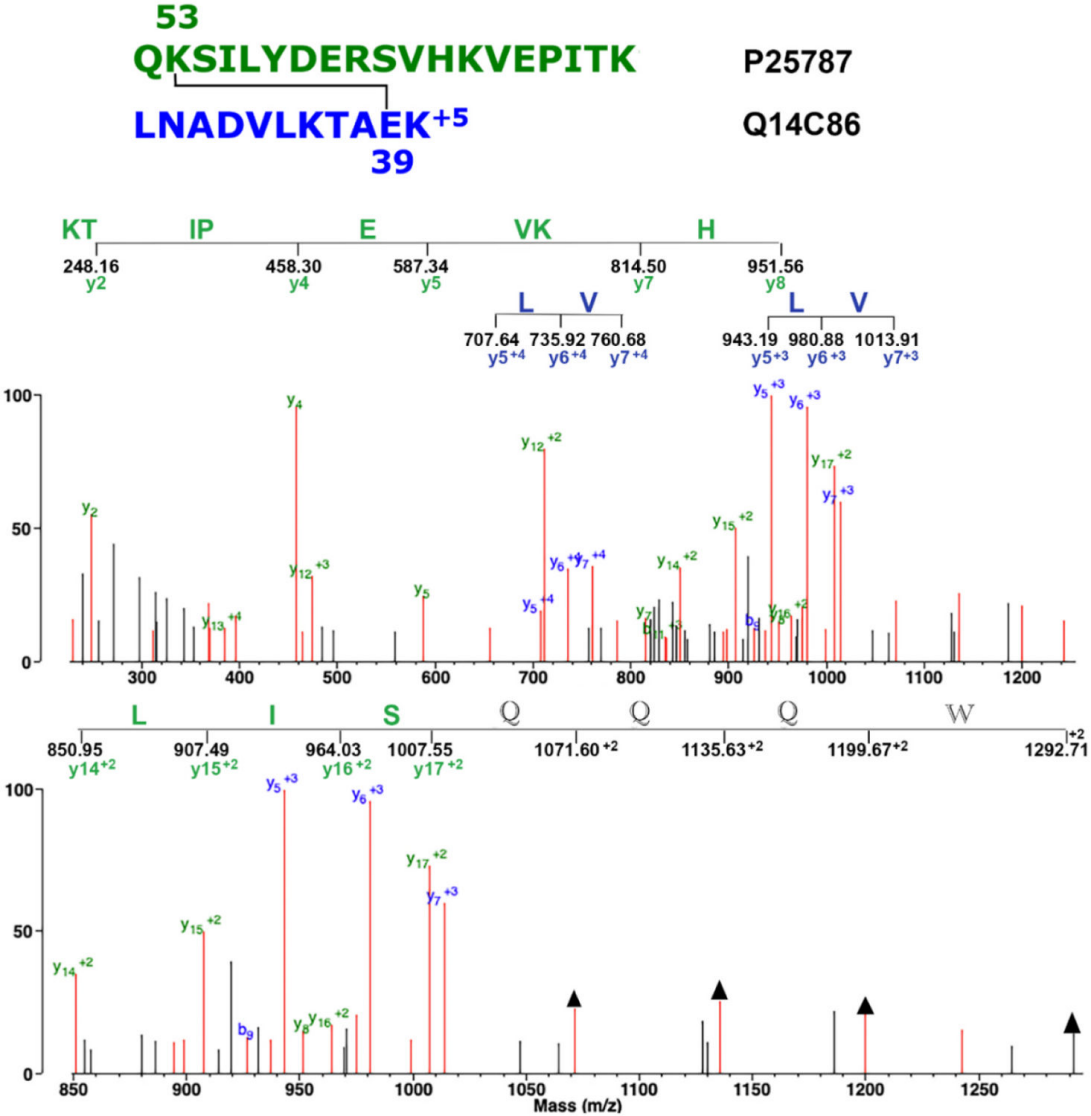
The MS/MS spectrum for the isopeptide crosslinked peptide QK_1062_SILYDERSVHKVEPITK/LNADVLKTAE_39_K, where the subscripted residues indicate the site of the isopeptide crosslink. The parent ion is at *m/z* 691.38, +5 charged. The upper panel shows the complete mass range *m/z* 180 to 1700. This shows an 8 amino acid, +1, y-ion sequence (HKVEIPTK) from the C-terminus of the green peptide; a 2-amino acid, +4, y-ion crosslink specific sequence (LV) from the blue peptide; and a repeat of that 2-amino acid crosslink specific sequence (LV) from the blue peptide in the +3 charge state. The lower panel shows an expanded mass range from *m/z* 850 to 1300. This emphasizes a 7-amino acid, +2 sequence (WQQQSIL) that is not consistent with the crosslinked peptide. Positions of the WQQQ masses are marked with arrows. Unlabeled, red masses mostly represent loss of water, amine, or CO.

**Fig. 5.**
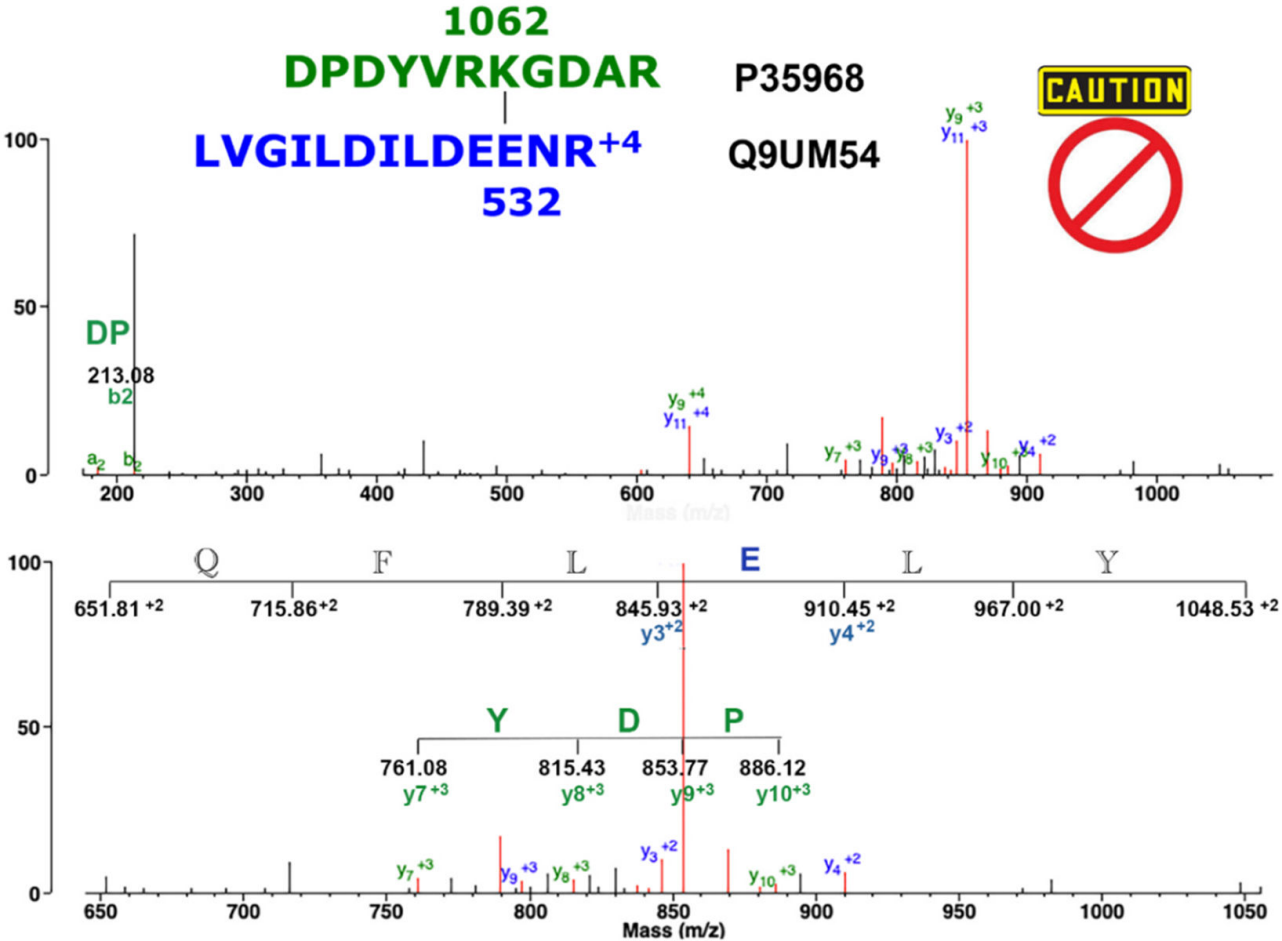
The MS/MS spectrum for the rejected isopeptide crosslinked peptide DPDYVRK_1062_GDAR/LVGILDILDEE_532_NR. The parent ion is at *m/z* 693.61, +4 charged. The upper panel shows the complete mass range from *m/z* 160 to 1100. There are no prominent crosslink features present. The lower panel shows an expanded mass range from 650 to *m/z* 1050. This emphasizes a 3-amino acid, +3, y-ion crosslinked specific sequence (PDY), and a 6-amino acid, +2 sequence QFLELY. The latter is inconsistent with the crosslink peptide but is consistent with kinesin family member 21A isoform CRA. C (A0A024R0V9). Unlabeled, red masses mostly represent loss of water, amine, or CO.

Figs. 2-5 illustrate the important features in the MS/MS spectra of isopeptide crosslinked peptides. The important features are detailed in Section 2.11. The fragmentation patterns in Figs. 2 and 3 show strong evidence to support a crosslinked peptide. Fig. 4 shows a minimally acceptable spectrum. Fig. 5 illustrates an unacceptable spectrum. Three additional MS/MS spectra with strong support for a crosslinked peptide are shown in the Supplemental material (Figs. S1, S2, & S3).

Fig. 2 shows the MS/MS spectrum of isopeptide crosslinked peptide AVNKVKDTPGLGK_450_VK/KGVDIE_4391_ISHR from the first replicate. Peptides are from kinesin-like protein KIF26B and hemicentin-1, respectively. Four crosslinked features are present that qualify the crosslink candidate to be a crosslinked peptide. Details of the fragmentation pattern are described in the figure legend.

Fig. 2 Panel A illustrates the N-terminal sequences from the blue and green peptides, and a ladder sequence, KGV, from the shorter, blue peptide. The ladder sequence fragments are crosslink specific ions. Fig. 2 Panels B and C illustrate mixed fragmentation. This version of mixed fragmentation can be envisioned to occur in two ways.

The first scenario is illustrated in Fig. 2 Panel B. Fragmentation is initiated by formation of y8^+2^ from the blue peptide AVNKVKDTPGLGK_450_VK/VDIE_4391_ISHR, *m/z* 1252.21. This fragment loses the N-terminal V from the blue peptide to make y7^+2^ AVNKVKDTPGLGK_450_VK/DIE_4391_ISHR, *m/z* 1202.68. That fragment undergoes a rearrangement that moves AVN from the N-terminal of the green peptide to the C-terminal. This results in the fragment KVKDTPGLGK_450_VKAVN/DIE_4391_ISHR that still has a molecular weight of *m/z* 1202.68. That is, there are two equivalent structure for the *m/z* 1202.68 mass. Sequential removal of KVK from the N-terminal of the green peptide would yield the remaining fragments VKDTPGLGK_450_VKAVN/DIE_4391_ISHR, *m/z* 1138.64, KDTPGLGK_450_VKAVN/DIE_4391_ISHR, *m/z* 1089.10, and DTPGLGK_450_VKAVN/DIE_4391_ISHR, *m/z* 1025.06.

The second scenario is illustrated in Fig. 2, Panel C. Fragmentation is initiated by formation of fragments y8^+2^ and y7^+2^, in the way described for the first scenario. The blue y7^+2^ mass at *m/z* 1202.68 for fragment AVNKVKDTPGLGK_450_VK/DIE_4391_ISHR is coincidently the same mass as the green y12^+2^ KVKDTPGLGK_450_VK/KGVDIE_4391_ISHR. This makes it appear as if green KVK is contiguous with the blue V, when it is not. Rather there are two separate fragmentation processes. Sequential removal of KVK from the N-terminal of the green peptide would yield the remaining fragments y11^+2^ VKDTPGLGK_450_VK/KGVDIE_4391_ISHR, y10^+2^ *m/z* 1138.64, KDTPGLGK_450_VK/KGVDIE_4391_ISHR, *m/z* 1089.10, and y9^+2^ DTPGLGK_450_VK/KGVDIE_4391_ISHR *m/z* 1025.06.

Either of these mixed fragmentation scenarios supports the same isopeptide crosslinked peptide.

Assignment of the crosslink to K450-E4391 is based on the following logic. The crosslinks we are investigating are limited to KE and KD. The crosslinked peptides AVNKVKDTPGLGK450VK (green peptide)/KGVDIE4391ISHR (blue peptide) together contain one E, two D, and five K residues. K4386 in the smaller, blue peptide is a b1 ion and is not a crosslink specific ion. Because there are no other K residues in the blue peptide, K in the isopeptide crosslink cannot come from the blue peptide. Four K residues in the green peptide are potential partners. K441 and K443 are released from the green peptide in panel B to make crosslinked fragments at 1138.64 and 1025.06. Since the peptides remain crosslinked when K441 and K443 are gone, K441 and K443 cannot be crosslink partners. K452 is at the C terminus, which was the cleavage site for trypsin. As a general rule, trypsin does not cleave at modified lysine residues therefore K452 cannot be a crosslink partner. This leaves K450 as the only possible lysine crosslink partner in the green peptide.

Fig. 3 shows the MS/MS spectrum for the isopeptide crosslinked peptide DVK_245_PSNILLDER/HLAESKE_927_K. Peptides are from dual specificity mitogen-activated protein kinase kinase 7 and ankyrin repeat domain-containing protein 12, respectively. Four crosslinked features are present that qualify the crosslink candidate to be a crosslinked peptide. Details of the fragmentation pattern are described in the figure legend.

Fig. 4 shows the MS/MS spectrum for the isopeptide crosslinked peptide QK_1062_SILYDERSVHKVEPITK/LNADVLKTAE_39_K. Peptides are from proteasome subunit alpha type-2 and GTPase-activating protein and VPS9 domain-containing protein 1, respectively. Three crosslinked features are present that qualify the crosslink candidate to be a crosslinked peptide. Plus, there is a 7-amino acid, +2 sequence (LISQQQW) that is not consistent with the crosslinked peptide. Peptide LISQQQW does not disqualify the crosslink because the crosslink is adequately supported by the green KTIPEVKH peptide and the blue crosslink specific ions for peptide LV. Details of the fragmentation pattern are described in the figure legend.

Fig. 5 shows the MS/MS spectrum for dipeptide DPDYVRK_1062_GDAR/LVGILDILDEE_532_NR that does not qualify as an isopeptide crosslinked peptide. Peptides are from vascular endothelial growth factor receptor 2 and unconventional myosin-VI, respectively. There is only one crosslinked feature in the spectrum, the green PDY fragment. The 6-amino acid, +2 sequence (QFLELY) is not consistent with the crosslink candidate. Unassigned amino acids appended to the blue E in peptide QFLELY disqualify E as support for the blue peptide. This removes support for the blue peptide. Without support for both peptides, the candidate crosslink in Fig. 5 is rejected. Details of the fragmentation pattern are described in the figure legend.

## 4. Discussion

### 4.1. Isopeptide crosslinks induced by other organophosphates

In previous studies we have used the organophosphate pesticide chlorpyrifos oxon to induce isopeptide crosslinks in the pure proteins butyrylcholinesterase, casein, serum albumin and tubulin [5,6]. Schmidt et al. found that the organophosphorus nerve agent VX induced crosslinks in ubiquitin [7]. The results from the current work demonstrate that the organophosphate pesticide dichlorvos can also induce isopeptide crosslinks. From these observations it is tempting to suggest that any organophosphylate may be capable of inducing isopeptide crosslinks in a variety of proteins.

### 4.2. Mechanism for the reaction of organophosphates with lysine

In a previous study [6] we showed that the organophosphate chlorpyrifos oxon reacted with lysine residues in bovine casein, human serum albumin, mouse serum albumin, human butyrylcholinesterase, and porcine tubulin to form diethylphospho-adducts. Only selected lysine residues were labeled, suggesting that reactive lysines were activated. It was proposed that activation involved through-space, charge-charge interactions with nearby negatively charged residues. Consistent with this proposal, half of the reactive lysine residues were within two residues of an acidic residue in the linear sequence. Support for subsequent OP-induced isopeptide bond formation was the observation that 77% of the crosslinks detected involved a lysine that had been labeled with diethylphosphate.

### 4.3. Organophosphate induced isopeptide crosslinks and disease

Epidemiological evidence suggests that there is an increased incidence of Alzheimer’s disease [18,19] and Parkinson’s [20] disease in agricultural workers who are exposed to organophosphorus pesticides. A hallmark for both diseases is accumulation of aggregated protein. Previously, we demonstrated that chlorpyrifos oxon, the activated form of the pesticide chlorpyrifos, can crosslink proteins [6]. Of the proteins we have studied in vitro, tubulin is the most sensitive to reaction with chlorpyrifos oxon. Reaction leads to formation of aggregates [9]. Treatment of mice with nonlethal levels of chlorpyrifos resulted in disruption of microtubule structures in the brain [21]. In light of the critical role that microtubules play, disruption of their structure would be expected to presage neurological problems. More recently, we have shown that chlorpyrifos oxon can induce isopeptide dimerization of amyloid beta (1–42) [22]. The covalent amyloid beta dimer is considered to be the toxic form of amyloid beta that leads to protein aggregation and disease. It follows that exposure to organophosphorus pesticides could contribute to the progression of diseases associated with aggregation. At this point, we have no data on how common protein crosslinking is upon exposure to organophosphate levels encountered in the environment, however, because development of Alzheimer’s and Parkinson’s disease appears to require years, slow accumulation of organophosphorus-induced aggregates could be a causative factor.

### 4.4. Compare isopeptide crosslinks created by transglutaminase to those induced by organophosphates

Transglutaminase is well known for creating γ-glutamyl-ε-lysine isopeptide crosslinks between an α-carboxy group from glutamine on one protein and an ε-amino group from lysine on another. In 2007 Kang et al. reported that isopeptide bonds could form spontaneously between the side chains of lysine and asparagine on Spy0128 in the polymeric shaft of pili expressed by S. pyogenes [8,23]. More recently, it has been reported that organophosphylates can promote spontaneous isopeptide formation between the side chains of lysine and glutamate or lysine and aspartate [6,7]. The consequence of isopeptide bond formation by any of these methods is the creation of protein dimers and higher oligomers. Dimers and molecular aggregates created by transglutaminase are associated with neurodegenerative diseases [24,25].

### 4.5. The process of identifying isopeptide crosslinks

Identifying isopeptide crosslinks in mass spectral data is technically challenging and somewhat subjective. In the Methods section we outlined a procedure for selecting isopeptide crosslinked peptides from raw mass spectral data. The process begins with a database search of the mass spectral data against a SwissProt database using the Protein Prospector/Batch Tag Web algorithm (see the Methods section Batch Tag Web search for crosslinked peptide candidates). Such searches generate large numbers of crosslink candidates that can be listed using Protein Prospector/Search Compare. This list is then reduced by manually screening the crosslink candidates against parameters generated by Protein Prospector that reflect the probability that crosslinked peptide assignment is correct. Parameters used in this work are charge state 3, 4, or 5; Score >25; score difference >1; % matched intensity >45%; and a minimum of 5 amino acids in each peptide. These parameters were arrived at through experience. They reduce the number of crosslink candidates to a manageable value. MS/MS spectra for the resultant crosslink candidates are manually evaluated for features that are indicative of an isopeptide crosslink (see the Methods section Manual evaluation of crosslinked peptides).

**Fig. 6.**
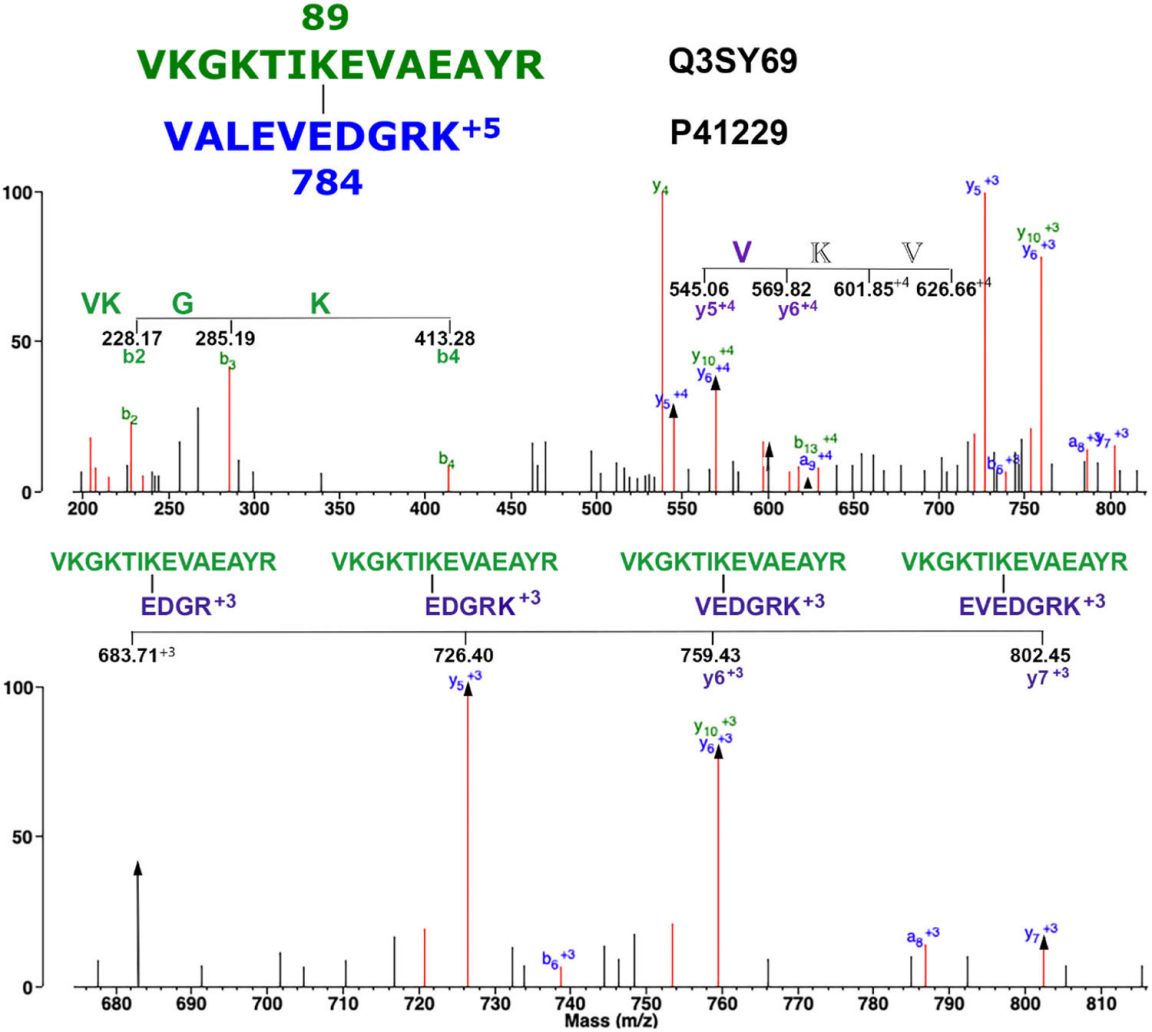
The MS/MS spectrum for the isopeptide crosslinked peptide VKGKTIK_89_EVAEAYR/VALEVE_784_DGRK. The parent ion is at *m/z* 538.517, +5 charged. The upper panel shows the mass range from *m/z* 200 to 800. This emphasizes a 4-amino acid, +1, b-ion sequence (VKGK) from the N-terminus of the green peptide and a 3-amino acid sequence (VKV) that contains 2 residues that cannot be assigned to the crosslink peptide sequence thereby disqualifying this feature as support for the crosslinked peptide. The lower panel shows a mass range from *m/z* 660 to 840. This emphasizes a 3-amino acid, +3, mixed fragmentation sequence (EVK) from the blue peptide. Mixed fragmentation peaks are marked with arrows. Unlabeled, red masses mostly represent loss of water, amine, or CO.

Though this process works, it relies on subjective decision. For example, we found that Protein Prospector can assign different crosslink candidates to the same MS/MS spectrum. Parent ion 538.517 mass (+5) appeared four times in the third replicate with elution times of 29.12, 29.63, 100.27, and 100.77 min. The MS/MS pattern was essentially the same for all four. Protein Prospector assigned these candidates to peptide sequences VKGKTIK_89_EVAEAYR/VALEVE_784_DGRK (29.12 min); KRRAQEE_196_AK/VKGKTIK_89_EVAEAYR (29.63 min); KGVQLQT HPNVDK_335_K/QRVKKDVD_260_K (100.27 min) AVNKVKDTPGLGK_450_VK/KGVDIE_4391_ISHR (100.77 min). The first, second, and third sequences each have two crosslink features in their MS/MS spectra, one for each peptide. The fourth sequence has four crosslink features in its MS/MS spectrum. Therefore, all four candidates have enough crosslink features to qualify as crosslinked peptides. Fig. 6 shows the MS/MS spectrum for the *m/z* 538.517 isopeptide crosslinked peptide VKGKTIK_89_EVAEAYR/VALEVE_784_DGRK. Details of the fragmentation pattern are described in the figure legend.

**Fig. 7.**
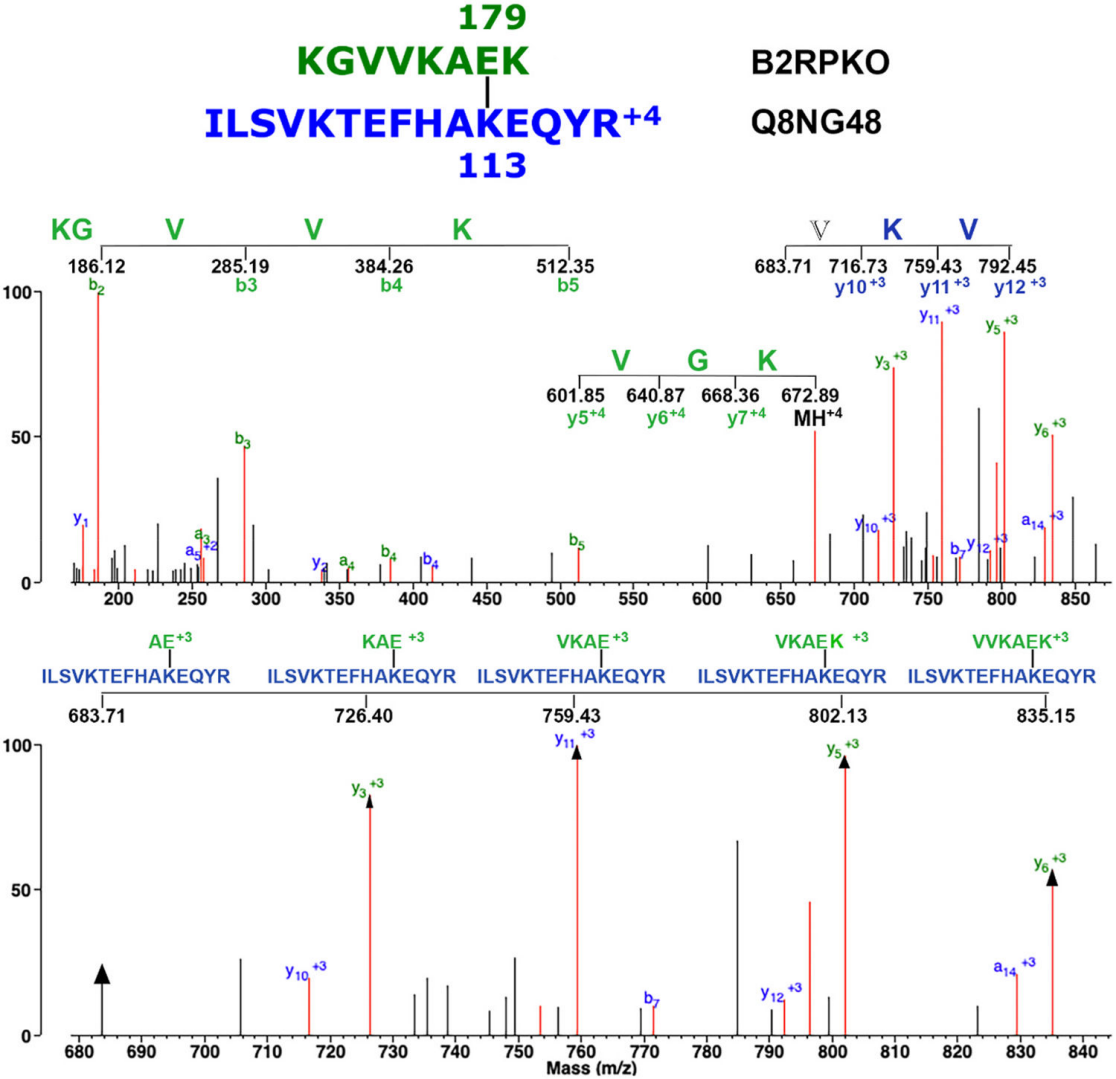
The MS/MS spectrum for the isopeptide crosslinked peptide KGVVKAE_179_K/ISLVKTEFHAK_113_EQYR. The parent ion is at *m/z* 672.894, +4 charged. The upper panel shows the mass range from *m/z* 150 to 870. This emphasizes a 5-amino, +1, b-ion sequence (KGVVK) from the N-terminus of the green peptide; a 3-amino acid, +4, ladder sequence from the N-terminus of the green peptide; and a 2-amino acid, +3, y-ion crosslink specific sequence (VK) from the blue peptide appended by an unassigned residue V. The lower panel shows the mass range from *m/z* 680 to 840. This emphasizes the mixed fragmentation sequence (VKVK) from the green peptide. Mixed fragmentation peaks are marked with arrows. Unlabeled, red masses mostly represent loss of water, amine, or CO.

The issue is further complicated by the fact that there is an *m/z* mass that is the +4 version of *m/z* 538.517. The *m/z* 672.894 mass also appeared four times in the third replicate with elution times of 29.11, 29.61, 31.56, and 100.80 min. Again, the MS/MS spectral pattern was essentially the same for all four, and the pattern was very similar to that for the *m/z* 538.517 masses. One might expect that Protein Prospector would assign the same crosslink candidate sequences to the *m/z* spectra that it did to the *m/z* 538.517 spectra. This is not the case. Protein Prospector assigned these candidates to peptide sequences KGVVKAE_179_K/ISLVKTEFHAK_113_EQYR (29.11 min); QRKVVKHP K_113_R/GKLKNVQSE_422_TKGLR (29.61 min); AVNKVKDTPGLGK_450_VK/KGVDIE_4391_ISHR (31.56 min); and GKVRVEK_2031_EK/VKNAVKYLQSLE_92_RS (100.80 min). Of these, only the third candidate is present in the *m/z* 538.517 group. The first and fourth sequences have four crosslinked features and therefore qualify as crosslinked peptides. The second and third sequences have five crosslink features and as such also qualify as crosslinked peptides. Fig. 7 shows the MS/MS spectrum for the *m/z* 672.894 isopeptide crosslinked peptide KGVVKAE_179_K/ISLVKTEFHAK_113_EQYR. Details of the fragmentation pattern are described in the figure legend.

Deciding which crosslinked sequence is correct is rather subjective. We chose AVNKVKDTPGLGK_450_VK/KGVDIE_4391_ISHR because it appeared in both the *m/z* 538.517 and 672.894 data sets, and because it appeared in the first and second replicates as well. It is noteworthy that the same sequence was assigned to the MS/MS spectrum for the *m/z* 896.86 (+3) parent ion in Fig. 2.

## 5. Conclusion

A protocol is presented for identifying zero-length isopeptide crosslinks in a complex protein mixture. Enrichment of tryptic peptides by binding to *anti*-isopeptide antibody is a key first step. Stringent washing of the immunopurified complex with a detergent-containing buffer minimizes the number of false positives. Searches of mass spectrometry data with Protein Prospector software provides a list of candidate dipeptide crosslinks. Manual evaluation of candidate crosslinked peptides is laborious but critical.

Our protocol identified isopeptide crosslinked proteins in cultured cells that had been exposed to 10 μM dichlorvos. Isopeptide crosslinks induced by organophosphate pesticides are distinct from crosslinks induced by transglutaminase. The chemically induced crosslinks are between lysine and glutamic acid or lysine and aspartic acid with release of a molecule of water. The transglutaminase induced crosslinks are between lysine and glutamine with release of a molecule of ammonia.

Protein aggregates in neurodegenerative diseases are thought to be produced in part by the action of transglutaminase. Our results suggest that exposure to organophosphorus pesticides may also be implicated.

## Supplementary Material

Supplementary Material

## Figures and Tables

**Table 1:** Confirmed crosslinked peptides.

#	Protein	UniProt ID	Mass *m/z*	Elute Time	Z	Score + Score Diff^a^	% Match^b^	Peptides^c^
First Replicate								
1	Kinesin-like protein KIF26B	Q2KJY2	672.8934	22.88	4	47.4 + 1.9	82.7	AVNKVKDTPGLGK_450_VK
	Hemicentin-1	Q96RW7						KGVDIE_4391_ISHR
2	Kinesin-like protein KIF26B	Q2KJY2	896.8553	22.95	3	34.2 + 2.4	76.8	AVNKVKDTPGLGK_450_VK
	Hemicentin-1	Q96RW7						KGVDIE_4391_ISHR
3	Dual specificity mitogen-activated protein kinase kinase 7	O14733	774.4123	26.84	3	29.2 + 8.4	51.8	DVK_245_PSNILLDER
	Ankyrin repeat domain-containing protein 12	Q6UB98						HLAESKE_927_K
4	Supervillin	O95425	835.1508	24.16	3	22.9 + 1.7	67.7	VKVRE_308_K
	UDP-GlcNAc:betaGal beta-1,3-N-acetylglucosaminyl transferase-like protein 1	Q67FW5						KVVAFCDVDENK_298_IRK
5	Transcription factor 20	Q9UGU0	626.6151	24.08	4	56.6 + 9.1	84.6	VKVRHK_1754_SASNGSK
	Myosin-4	Q9Y623						VKKQLDHE_1540_K
6	Serine/threonine-protein kinase WNK2	Q9Y3S1	831.2604	24.25	4	43.2 + 3.4	82.5	TVGAAQLK_2180_PTLNQLKQTQK
	Oxysterol-binding protein-related protein 5	Q9H0X9						VTKKE_97_TLKAQK
7	Tubulin polyglutamylase TTLL7	Q6ZT98	673.1405	23.27	4	34.2 + 4.1	57.0	QRKVLDIVK_742_TSIR
	Transcription elongation factor A protein-like 2	Q9H3H9						KGKSE_83_MoxQGGSK
8	Gem-associated protein 4	P57678	558.1219	23.35	5	54.6 + 1.6	67.9	NK_497_VLAGILRSWGRK
	Lysosomal-trafficking regulator	Q99698						QD_199_HPKAKLDR
Second Replicate								
9	Kinesin-like protein KIF26B	Q2KJY2	672.8947	24.04	4	52.6 + 4.4	81.1	AVNKVKDTPGLGK_450_VK
	Hemicentin-1	Q96RW7						KGVDIE_4391_ISHR
10	Microcephalin	Q8NEM0	538.5172	24.111	5	39.1 + 1.9	69.0	GKKPTRTLVMoxTSMPSE_658_K
	Junction-mediating and -regulatory protein	Q8N9B5						KGVKLK_936_K
11	Centromere protein V-like protein 3	A0A0U1RRI6	626.6141	24.54	4	52.5 + 12.5	83.1	VQVGVGSHAAAK_67_RWLGK
	Fibroblast growth factor receptor-like 1	Q8N441						VKQVE_90_R
12	Elongation factor 2	P13639	704.9792	26.01	5	50.5 + 16.7	64.3	K_16_ANIRNMSVIAHVDHGK
	Protein eva-1 homolog A	Q9H8M9						NVFTSAE_116_ELERAQR
13	Proteasome subunit alpha type-2	P25787	691.3914	21.50	5	54.2 + 24.6	65.1	QK_53_SILYDERSVHKVEPITK
	GTPase-activating protein and VPS9 domain-containing protein 1	Q14C86						LNADVLKTAE_39_K
14	Transcriptional regulator ATRX	P46100	820.4745	26.86	4	44.2 + 4.2	65.0	ENLLGSIK_1774_EFR
	Isoleucine-tRNA ligase, cytoplasmic	P41252						YPLKEIVVIHQD_856_PEALK
15	Prohibitin-2	Q99623	811.0315	24.93	5	36.0 + 8.6	46.0	QK_224_IVQAEGEAEAAK
	Probable 2-oxoglutarate dehydrogenase E1 component DHKTD1, mitochondrial	Q96HY7						TLVFCSGKHFYSLVKQRE_821_SLGAK
16	Oxysterol-binding protein-related protein 5	Q9H0X9	831.0162	26.55	4	36.1 + 2.1	68.3	VTKKE_97_TLKAQK
	Serine/threonine-protein kinase WNK2	Q9Y3S1						TVGAAQLK_2180_PTLNQLKQTQK
Third Replicate								
17	Kinesin-like protein KIF26B	Q2KJY2	538.5171	100.77	5	37.7 + 2.8	65.4	AVNKVKDTPGLGK_450_VK
	Hemicentin-1	Q96RW7						KGVDIE_4391_ISHR
18	Mitochondrial 10-formyltetrahydrofolate dehydrogenase	Q3SY69	538.5169	29.11	5	29.0 + 7.4	57.2	VKGKTIK_89_EVAEAYR
	Lysine-specific demethylase 5C	P41229						VALEVE_784_DGRK
19	DnaJ homolog subfamily C member 9	Q8WXX5	538.5166	29.63	5	43.5 + 1.0	69.0	KRRAQEE_196_AK
	Mitochondrial 10-formyl tetrahydrofolate dehydrogenase	Q3SY69						VKGKTIK_89_EVAEAYR
20	Coatomer subunit delta	P48444	538.5165	100.27	5	26.5 + 0	58.7	KGVQLQTHPNVDK_335_K
	Kelch domain-containing protein 4	Q8TBB5						QRVKKDVD_260_K
21	Kinesin-like protein KIF26B	Q2KJY2	672.8947	31.55	4	31.7 + 4.1	69.3	AVNKVKDTPGLGK_450_VK
	Hemicentin-1	Q96RW7						KGVDIE_4391_ISHR
22	Ankyrin −2	Q01484	672.8947	100.84	4	24.3 + 1.6	73.1	GKVRVEK_2031_EK
	C─C motif chemokine 17	Q92583						VKNAVKYLQSLE_92_RS
23	Putative high mobility group protein B1-like 1	B2RPK0	672.8946	29.11	4	40.0 + 10.2	53.3	KGVVKAE_179_K
	Protein Lines homolog 1	Q8NG48						ISLVKTEFHAK_113_EQYR
24	Osteocrin	P61366	672.8939	29.61	4	48.6 + 10.5	77.9	QRKVVKHPK_113_R
	Helicase-like transcription factor	Q14527						GKLKNVQSE_422_TKGLR
25	Titin	Q8WZ42	626.6149	29.44	4	43.1 + 10.8	78.6	VKVQD_28977_TPGK
	Alpha-1,3-mannosyl-glycoprotein 4-beta-N-acetylglucosaminyl transferase-like protein MGAT4D	A6NG13						VLNRMK_71_YEITKR
26	StAR-related lipid transfer prot 9	Q9P1P6	671.7452	25.80	3	33.5 + 3.0	61.8	RKKVSFQLE_773_R
	Collagen alpha-5(VI) chain	A8TX70						KGVK_1667_GPR
27	Zinc finger protein 292	O60281	896.8561	29.75	3	32.5 + 4.7	69.1	KRKVE_2548_K
	Arginase-2, mitochondrial	P78540						KGVEHGPAAIREAGLMK_56_R
28	WD repeat-containing protein 1	O75083	872.0002	28.52	4	29.9 + 0.2	62.8	VFASLPQVE_16_RGVSKIIGGDPK
	DNA repair protein RAD50	Q92878						STESELKKK_722_EK
29	Zinc finger protein 728	P0DKX0	928.9985	28.54	4	46.4 + 6.6	65.1	AIHAGEKLYK_481_CEECGK
	Uncharacterized protein C9orf43	Q8TAL5						MFLSIHRLTLE_269_RPALR
30	Lysine-specific demethylase 7A	Q6ZMT4	736.4008	33.85	5	33.6 + 5.4	63.5	FNKHLQPSSTVPE_569_WRAKDNDLR
	Inter-alpha-trypsin inhibitor heavy chain H2	P19823						VQISVKK_810_EK
31	Complement receptor type 2	P20023	825.7779	26.33	3	28.0 + 4.9	51.3	LIGEKSLLCITK_68_DK
	Oxysterol-binding protein-related protein 9	Q96SU4						D_680_IDAATEAK
32	14-3-3 protein theta	P27348	758.4285	33.48	4	44.0 + 1.0	66.3	NLLSVAYK_49_NVVGGRR
	Interleukin-10 receptor subunit beta	Q08334						NILQWE_41_SPAFAK
33	14-3-3 protein theta	P27348	916.2504	32.67	4	39.1 + 5.7	56.8	NLLSVAYK_49_NVVGGRRSAWR
	Serologically defined colon cancer antigen 8	Q86SQ7						QVLQISEE_349_ANFEK
34	von Willebrand factor A domain-containing protein 3B	Q502W6	591.5668	27.80	4	27.2 + 9.0	50.2	D_794_GLSNASSRR
	Phosphatidylinositol 4-kinase alpha	P42356						IHNELSPLK_432_LR
35	Coatomer subunit alpha	P53621	606.9457	33.54	5	41.3 + 6.1	61.1	VTTVTEIGK_1211_DVIGLR
	Teneurin-4	Q6N022						NLLSLDFD_1844_RVTR
36	Microfibrillar-associated protein 1	P55081	835.1493	29.59	3	27.6 + 3.4	61.6	VKVK_37_RYVSGK
	Serine/threonine-protein kinase SMG1	Q96Q15						KVVD_1635_NASQGEGVR
37	14-3-3 protein zeta/delta	P63104	916.0831	34.06	5	61.4 + 5.3	80.9	SVTEQGAELSNEERNLLSVAY
	Striated muscle preferentially expressed protein kinase	Q15772						K_49_NVVGAR RLFQQKAASLDE_538_R
38	Staphylococcal nuclease domain -containing protein 1	Q7KZF4	847.6662	36.73	5	58.8 + 18.8	65.7	QFQK_873_VITEYLNAQESAK
	Zinc finger protein 777	Q9ULD5						LAVWAAVQAVE_196_RKLEAQA MoxR
39	C-type lectin domain family 4 member M	Q9H2X3	537.6976	27.08	5	37.0 + 2.9	56.8	LQEIYQELTE_210_LK
	Betaine-homocysteine S-methyltransferase 1	Q93088						ELFEKQK_402_FK
40	Teneurin-3	Q9P273	762.4280	31.48	4	50.0 + 2.2	74.4	NLLSVD_1778_FDRTTKTEK
	Rho guanine nucleotide exchange factor 26	Q96DR7						RALKEVSK_615_LVR

a Score and score difference values are assigned by Protein Prospector. The higher the value, the better the match to a theoretical peptide. In Table 1, values range from 24.3 to 61.4.

b % match is the matched intensity of the assigned peaks compared to the observed peaks. Confidence in the assignment is associated with high % matched intensity.

c Crosslinked residues are suffixed by a subscripted number identifying the crosslinked residue.

## Data Availability

Data are deposited in the PRIDE database. Identifier PXD033593. Reviewer account details: Username reviewer_pxd033593@ebi.ac.uk; password Fk7nQocd.

## Abbreviations

LC-MS/MS: liquid chromatography tandem mass spectrometry
MS/MS: tandem mass spectral fragmentation
DCV: dichlorvos
iBAQ: intensity based absolute quantitation
PBS: phosphate buffered saline
SDS: sodium dodecyl sulfate
SH-SY5Y: human neuroblastoma cells
