# Supplementary material for: Dichlorvos-induced formation of isopeptide crosslinks between proteins in SH-SY5Y cells
Source: Anal Biochem. Author manuscript; Available in PMC 2023 Jul 25. (PMC10368009; doi:10.1016/j.ab.2022.114844)
Supplement: Supplementary Material [file NIHMS1916538-supplement-Supplementary_Material.docx]

Supplementary Material

for

Dichlorvos-induced formation of isopeptide crosslinks between proteins in SH-SY5Y cells

by

LM Schopfer & O Lockridge

**Table S2**

Protein Abundance ^a^

| **Protein** | **UniProt**  **ID** | **Copy/cell ^b^**  **Aebersold**  **U20S** | **iBAQ ^c^**  **Mann**  **A549 GAMG HEK293 HeLa HepG2 Jurkat K562 LnCap MCF7 RKO U20S** |
| --- | --- | --- | --- |
| First Replicate | | | |
| Kinesin-like protein KIF26B  Hemicentin-1 | Q2KJY2  Q96RW7 | na  na | na na na na na na na na na na na  na na na na na na na na na na na |
| Kinesin-like protein KIF26B  Hemicentin-1 | Q2KJY2  Q96RW7 | na  na | na na na na na na na na na na na  na na na na na na na na na na na |
| Dual specificity mitogen-activated protein kinase kinase 7  Ankyrin repeat domain-containing protein 12 | O14733  Q6UB98 | 2.27e3  na | 4.70 na 5.83 5.55 na 5.75 5.51 5.47 5.31 5.11 5.17  na na na na na na na na na na na |
| Supervillin  UDP-GlcNAc:betaGal beta-1,3-N-acetylglucosaminyl transferase-like protein 1 | O95425  Q67FW5 | 3.86e3  na | 5.10 4.21 5.51 5.18 4.35 4.80 na 4.46 5.27 3.79 5.50  na 4.64 na 4.37 4.40 5.15 4.27 5.42 na na 4.40 |
| Transcription factor 20  Myosin-4 | Q9UGU0  Q9Y623 | 1.83e3  na | 4.67 4.59 5.33 4.99 2.96 5.18 4.05 4.76 4.88 4.27 5.13  na na na na na na 2.68 na 4.44 4.13 na |
| Serine/threonine-protein kinase WNK2  Oxysterol-binding protein-related protein 5 | Q9Y3S1  Q9H0X9 | na  <5e2 | 2.42 3.64 4.27 na na na 4.16 3.95 na 4.40 na  4.19 5.35 4.44 4.68 na 5.55 na 518 5.03 na 5.20 |
| Tubulin polyglutamylase TTLL7  Transcription elongation factor A protein-like 2 | Q6ZT98  Q9H3H9 | na  4.43e3 | na na na na na na na na na na na  na na 3.62 na na na 6.98 na na na na |
| Gem-associated protein 4  Lysosomal-trafficking regulator | P57678  Q99698 | 5.74e3  na | 5.09 na 3.27 5.33 4.49 4.78 na 4.58 5.31 5.08 5.41  na 3.90 na na na 3.62 na na na na 3.54 |
| Second Replicate | | | |
| Kinesin-like protein KIF26B  Hemicentin-1 | Q2KJY2  Q96RW7 | na  na | na na na na na na na na na na na  na na na na na na na na na na na |
| Microcephalin  Junction-mediating and -regulatory protein | Q8NEM0  Q8N9B5 | na  na | na na 4.63 na na 4.27 4.52 na na na na  4.10 4.25 5.46 4.50 na 4.37 4.26 4.16 4.41 4.32 na |
| Centromere protein V-like protein 3  Fibroblast growth factor receptor-like 1 | A0A0U1RRI6  Q8N441 | na  na | na na na na na na na na na na na  na na na na na na na na na na na |
| **Protein** | **UniProt**  **ID** | **Copy/cell ^b^**  **Aebersold**  **U20S** | **iBAQ ^c^**  **Mann**  **A549 GAMG HEK293 HeLa HepG2 Jurkat K562 LnCap MCF7 RKO U20S** |
| Elongation factor 2  Protein eva-1 homolog A | P13639  Q9H8M9 | 4.42e6  na | 8.03 8.25 8.65 8.34 8.07 7.88 7.74 8.22 8.65 7.96 8.54  5.70 na na na na na na na na na 6.07 |
| Proteasome subunit alpha type-2  GTPase-activating protein and VPS9 domain-containing protein 1 | P25787  Q14C86 | 1.88e6  5.81e3 | 7.27 7.58 7.65 7.16 7.15 7.15 6.97 7.47 7.55 7.09 7.54  4.99 6.49 6.96 6.38 5.69 6.16 6.10 5.70 6.64 6.31 6.45 |
| Transcriptional regulator ATRX  Isoleucine--tRNA ligase, cytoplasmic | P46100  P41252 | <5e2  9.29e4 | 5.85 6.11 6.07 5.64 4.54 5.06 4.70 6.06 5.76 4.65 4.24  6.85 7.23 7.56 7.17 6.87 7.06 6.78 7.64 7.31 7.09 7.44 |
| Prohibitin-2  Probable 2-oxoglutarate dehydrogenase E1 component DHKTD1, mitochondrial | Q99623  Q96HY7 | 3.99e5  <5e2 | 7.81 7.77 8.02 7.92 7.73 7.22 7.13 8.04 7.97 7.57 7.86  5.13 5.31 6.16 5.39 4.36 5.35 5.05 5.54 6.70 4.98 4.96 |
| Oxysterol-binding protein-related protein 5  Serine/threonine-protein kinase WNK2 | Q9H0X9  Q9Y3S1 | <5e2  na | 4.19 5.35 4.42 4.86 na 4.58 na 5.55 5.03 na 5.20  2.42 3.64 4.27 na na na 4.16 3.95 na 4.40 na |
| Third Replicate | | | |
| Kinesin-like protein KIF26B  Hemicentin-1 | Q2KJY2  Q96RW7 | na  na | na na na na na na na na na na na  na na na na na na na na na na na |
| Mitochondrial 10-formyltetrahydrofolate dehydrogenase  Lysine-specific demethylase 5C | Q3SY69  P41229 | <5e2  <5e2 | 4.53 3.93 5.87 5.32 5.46 5.08 na na na na 513  4.52 5.62 5.24 4.27 3.91 4.33 4.56 4.77 5.11 4.32 4.87 |
| DnaJ homolog subfamily C member 9  Mitochondrial 10-formyl tetrahydrofolate dehydrogenase | Q8WXX5  Q3SY69 | 9.95e4  <5e2 | 6.70 6.68 7.15 6.92 6.18 7.15 6.72 6.65 7.27 6.54 7.55  4.53 3.93 5.87 5.32 5.46 5.08 na na na na 5.13 |
| Coatomer subunit delta  Kelch domain-containing protein 4 | P48444  Q8TBB5 | 1.46e5  7.97e3 | 6.44 7.38 7.59 7.13 7.18 6.79 6.61 7.68 7.64 6.69 7.43  5.26 4.96 6.11 5.73 3.85 5.71 5.49 5.16 6.58 5.29 5.47 |
| Kinesin-like protein KIF26B  Hemicentin-1 | Q2KJY2  Q96RW7 | na  na | na na na na na na na na na na na  na na na na na na na na na na na |
| Ankyrin -2  C-C motif chemokine 17 | Q01484  Q92583 | na  4.81e4 | na na na na na na na na na na 3.84  na na na na na na na na na na na |
| Putative high mobility group protein B1-like 1  Protein Lines homolog 1 | B2RPK0  Q8NG48 | na  na | 8.29 8.51 8.80 8.32 8.02 8.42 7.70 8.63 8.69 7.62 8.52  na na na na na na na na na na na |
| Osteocrin  Helicase-like transcription factor | P61366  Q14527 | na  3.00e3 | na na na na na na na na na na na  5.50 5.94 6.20 5.98 4.55 5.86 5.26 6.02 6.00 na 5.61 |
| Titin  Alpha-1,3-mannosyl-glycoprotein 4-beta-N-acetyl glucosaminyl transferase-like protein MGAT4D | Q8WZ42  A6NG13 | na  na | na na na na na na na na na na na  na na na na na na na na na na na |
| **Protein** | **UniProt**  **ID** | **Copy/cell ^b^**  **Aebersold**  **U20S** | **iBAQ ^c^**  **Mann**  **A549 GAMG HEK293 HeLa HepG2 Jurkat K562 LnCap MCF7 RKO U20S** |
| StAR-related lipid transfer protein 9  Collagen alpha-5(VI) chain | Q9P1P6  A8TX70 | na  na | na na na na na na na na na na na  na na na na na na na na na na na |
| Zinc finger protein 292  Arginase-2, mitochondrial | O60281  P78540 | na  3.49e3 | 3.68 5.76 3.57 5.60 na 4.49 3.72 3.17 5.22 3.66 3.23  na na 6.56 na 6.72 na 4.69 7.42 na 5.46 6.41 |
| WD repeat-containing protein 1  DNA repair protein RAD50 | O75083  Q92878 | 3.19e5  8.03e3 | 7.43 8.14 7.49 7.19 7.47 7.80 6.88 7.33 7.44 7.24 7.78  6.19 6.75 6.95 6.42 5.91 6.03 5.88 6.78 6.17 5.44 6.35 |
| Zinc finger protein 728  Uncharacterized protein C9orf43 | P0DKX0  Q8TAL5 | na  na | na na na na na na na na na na na  na na na na na na na na na na na |
| Lysine-specific demethylase 7A  Inter-alpha-trypsin inhibitor heavy chain H2 | Q6ZMT4  P19823 | na  na | na na na na na na na na na na na  5.68 5.46 4.90 5.33 6.53 6.25 5.36 5.36 4.65 4.79 5.24 |
| Complement receptor type 2  Oxysterol-binding protein-related protein 9 | P20023  Q96SU4 | na  1.23e4 | 7.24 7.82 7.57 7.36 7.55 7.17 6.64 7.82 7.67 6.30 7.14  5.55 6.56 6.71 5.97 5.39 5.77 5.48 5.42 6.15 5.13 6.43 |
| 14-3-3 protein theta  Interleukin-10 receptor subunit beta | P27348  Q08334 | 1.32e6  na | 8.04 8.13 8.04 7.70 7.50 7.72 7.01 7.90 7.98 7.25 8.21  na na na na na na na na na na na |
| 14-3-3 protein theta  Serologically defined colon cancer antigen 8 | P27348  Q86SQ7 | 1.32e6  <5e2 | 8.04 8.13 8.04 7.69 7.50 7.72 7.02 7.90 7.89 7.25 8.21  4.22 na 4.99 na na 4.59 4.40 na na nqa na |
| von Willebrand factor A domain-containing protein 3B  Phosphatidylinositol 4-kinase alpha | Q502W6  P42356 | na  <5e2 | na na na na na na na na na na na  4.86 5.52 5.58 4.37 4.51 4.67 5.32 5.40 5.55 4.12 5.47 |
| Coatomer subunit alpha  Teneurin-4 | P53621  Q6N022 | 2.21e5  na | 6.98 7.53 7.60 7.25 7.25 6.90 6.55 7.58 7.63 6.80 7.40  na na na na na na na na na na na |
| Microfibrillar-associated protein 1  Serine/threonine-protein kinase SMG1 | P55081  Q96Q15 | 6.04e3  6.00e2 | 5.88 6.36 6.82 6.59 5.43 6.27 5.82 6.28 5.74 5.26 6.27  4.70 5.31 5.25 4.74 4.89 4.79 4.56 5.38 5.37 4.72 5.07 |
| 14-3-3 protein zeta/delta  Striated muscle preferentially expressed protein kinase | P63104  Q15772 | 2.42e6  na | 8.46 8.66 8.61 8.26 8.08 8.09 7.91 8.34 8.51 7.92 8.76  na na na na na na na na na na 4.15 |
| Staphylococcal nuclease domain -containing protein 1  Zinc finger protein 777 | Q7KZF4  Q9ULD5 | 1.60e5  na | 7.33 7.70 7.69 7.34 7.13 7.05 7.04 7.67 7.55 7.09 7.55  na na na na na na na na na na na |
| C-type lectin domain family 4 member M  Betaine--homocysteine S‑methyltransferase 1 | Q9H2X3  Q93088 | na  na | na na na na na na na na na na na  na na na na na na na na na na na |
| Teneurin-3  Rho guanine nucleotide exchange factor 26 | Q9P273  Q96DR7 | <5e2  na | na 4.93 2.90 4.90 4.19 4.63 4.37 4.97 5.10 4.99 4.53  na 4.52 4.79 5.35 na 4.63 6.04 4.82 na 4.62 4.04 |
|  |  |  |  |

a na indicates that no value was found, implying that the protein abundance was too low to be detected.

b Protein abundance estimates for human osteosarcoma cell line (U20S) which contains 7311 proteins ranging in abundance from a maximum of 6.53x10^6^ copies per cell to a minimum of <5x10^2^ copies per cell[15]. Copy number was based on 144 heavy isotope labeled reference peptides.

c Protein abundance for representative examples from eleven cell lines (A549, GAMB, HEK293, HeLa, HepG2, K562, MCF7, Jurkat, LnCap, RKO, and U20S) which contain 11,732 proteins ranging in abundance from a iBAQ value of 2.41 to 9.13[16]. The iBAQ (intensity based absolute quantitation) is the value obtained by summing the peak intensities of all peptides matching to a specific protein and dividing by the number of theoretically observable peptides in the whole sample. It is reported as a log10 value[17]. Values are the average of three determinations.

Figure S1.


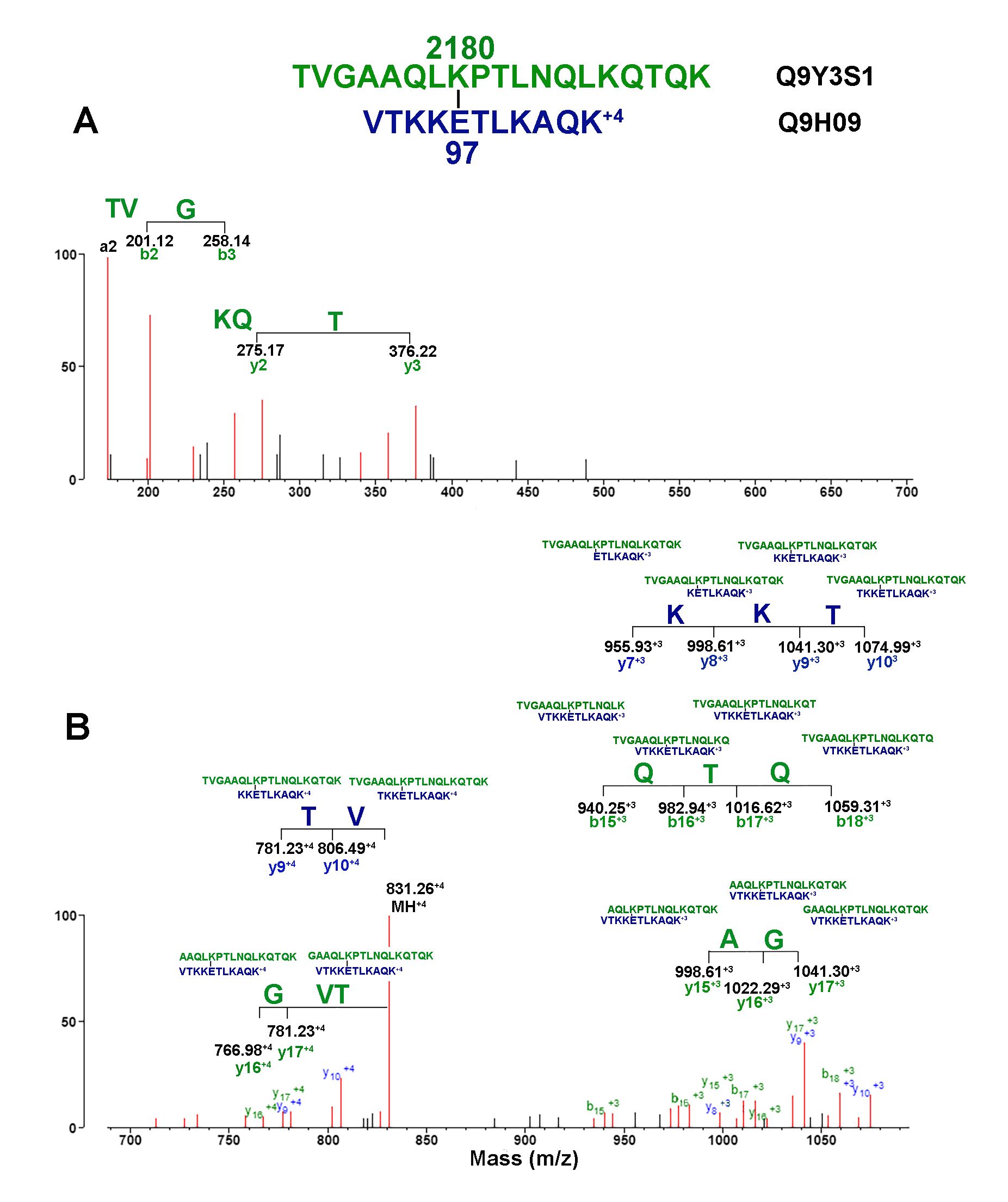


Fig. S1 The MS/MS spectrum for the isopeptide crosslinked peptide TVGAAQLK_2180_PTLNQLKQTQK/VTKKE_97_TLKAQK, where the subscripted residues indicate the site of the isopeptide crosslink. Peptides are from serine-threonine-protein kinase WNK2 and oxysterol-binding protein-related protein 5, respectively. The parent ion is at m/z 831.26, +4 charged. Panel A shows the mass range from m/z 180 to 700. This includes a 3-amino acid, +1, b-ion sequence (TVG) from the N terminal of the green peptide and a 3-amino acid, +1, y-ion sequence (KQT) from the green peptide. Panel B shows the mass range m/z 700 to 1090. This includes two ladder sequences (TVG) from the parent ion for the green peptide, and (VT) from the parent ion for the blue peptide. In addition, there are three crosslink specific sequences. There is a y-ion sequence (TKK) for the blue peptide, a b-ion sequence (QTQ) for the green peptide, and a y-ion sequence (GA) for the green peptide. Structures of the proposed fragments are shown. These ions give strong support for an isopeptide crosslinked peptide pair. Unlabeled, red masses mostly represent loss of water, amine, or CO.

Figure S2


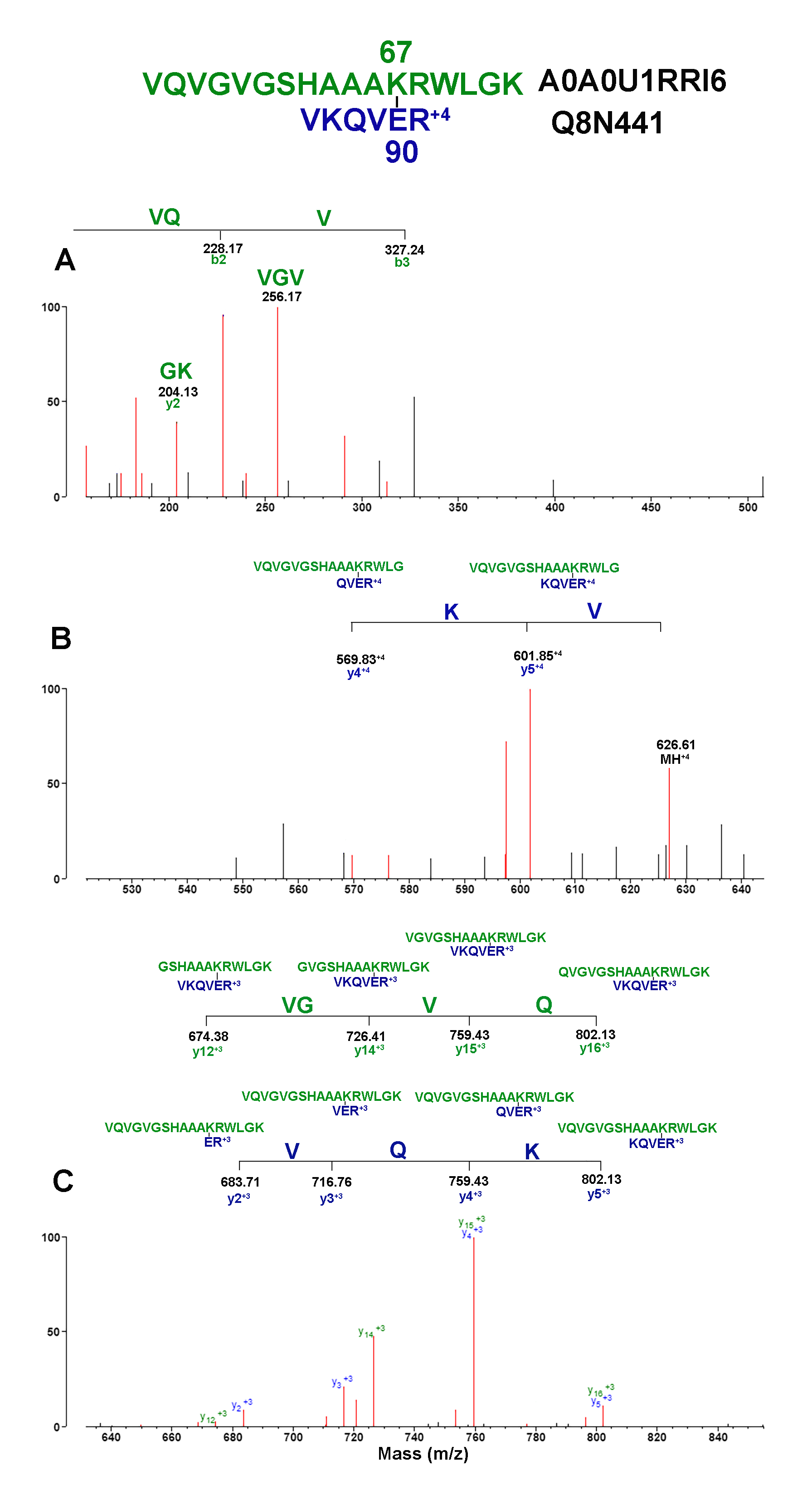


Fig. S2 The MS/MS spectrum for the isopeptide crosslinked peptide VQVGVGSHAAAK_67_RWLGK/VKVE_90_R, where the subscripted residues indicate the site of the isopeptide crosslink. Peptides are from centromere protein V-like protein 3 and fibroblast growth factor receptor-like 1, respectively. The parent ion is at m/z 626.61, +4 charged. Panel A shows the mass range from m/z 160 to 500. This includes a 3-amino acid, +1, b-ion sequence (VQV) from the N terminal of the green peptide, a 2-amino acid, +1, y-ion sequence (GK) from the C terminal of the green peptide and an internal fragment (VGV) from the green peptide. Panel B shows the mass range m/z 520 to 640. This includes a ladder sequence (VK) from the parent ion for the blue peptide. Note that the masses are also consistent with a ladder sequence (VQ) from the green peptide. Structures of the proposed fragments are shown. Panel C shows the mass range m/z 640 to 840. This includes two crosslink specific sequences. There is a y-ion sequence (KQV) for the blue peptide, and a y-ion sequence (QVGV) for the green peptide. These ions give strong support for the isopeptide crosslinked peptide pair. Structures of the proposed fragments are shown. Unlabeled, red masses mostly represent loss of water, amine, or CO.

Figure S3


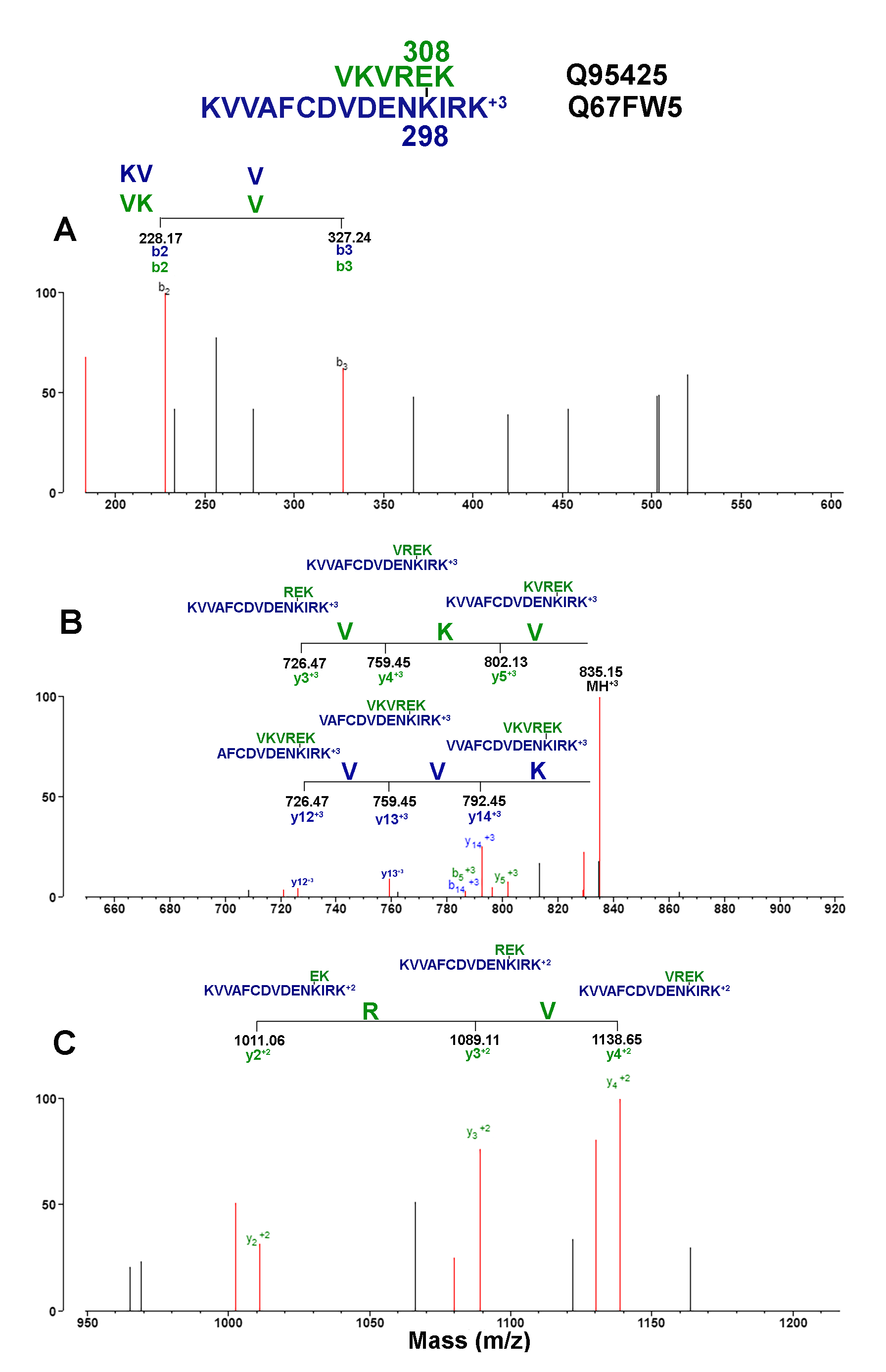


Fig. S3 The MS/MS spectrum for the isopeptide crosslinked peptide VKVRE_308_K/KVVAFCDVDENK_298_IRK, where the subscripted residues indicate the site of the isopeptide crosslink. Peptides are from supervillain and UDP-GlcNAc: beta Gal beta-1,3-N-aectylglucosaminyl transferase-like protein 1, respectively. The parent ion is at m/z 835.15, +3 charged. Panel A shows the mass range from m/z 190 to 600. This includes a 3-amino acid, +1, b-ion sequence (VKV) from the N terminal of the green peptide. Note that the same masses fit a b-ion sequence (KVV) from the N terminal of the blue peptide. Panel B shows the mass range m/z 640 to 920. This includes two ladder sequences (VVK) from the parent ion for the blue peptide, and (VKV) from the parent ion of the green peptide. Structures of the proposed fragments are shown. The ladder sequences are crosslink specific ions that strongly support the isopeptide crosslinked peptide pair. Panel C shows the mass range m/z 950 to 1200. This includes a crosslink specific y-ion sequence (RV) for the green peptide. Structures of the proposed fragments are shown. Unlabeled, red masses mostly represent loss of water, amine, or CO.
